# Supplementary figures and images for: Whole-genome scanning reveals environmental selection mechanisms that shape diversity in populations of the epipelagic diatom Chaetoceros
Source: PLoS Biol. 2022 Nov 28;20(11):e3001893. doi: 10.1371/journal.pbio.3001893 (PMC9731442; doi:10.1371/journal.pbio.3001893)

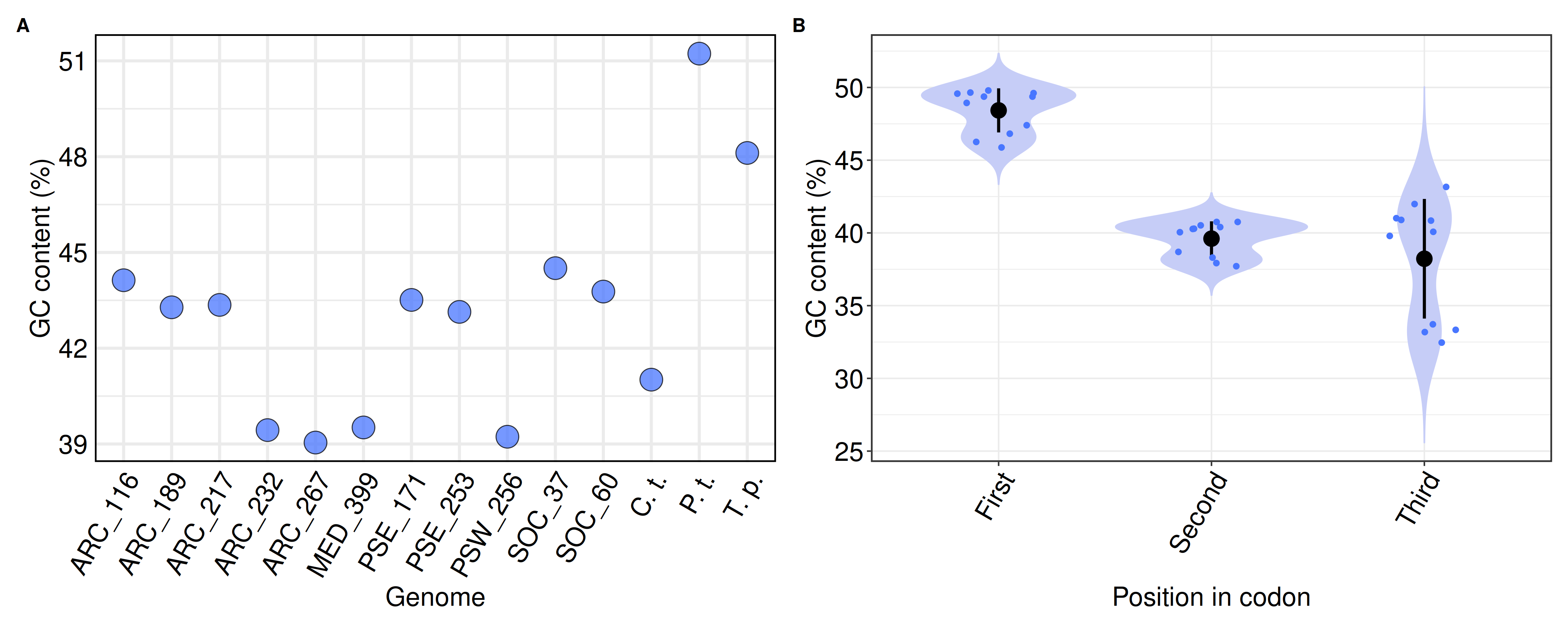

Supplement: S1 Fig — (A) Mean G+C content of MAGs and reference diatom genomes Chaetoceros tenuissimus (C.t.), P. tricornutum (P.t.), and T. pseudonana (T.p.). (B) Distribution of G+C content along codon positions of the MAGs. (PNG) [file pbio.3001893.s008.png]

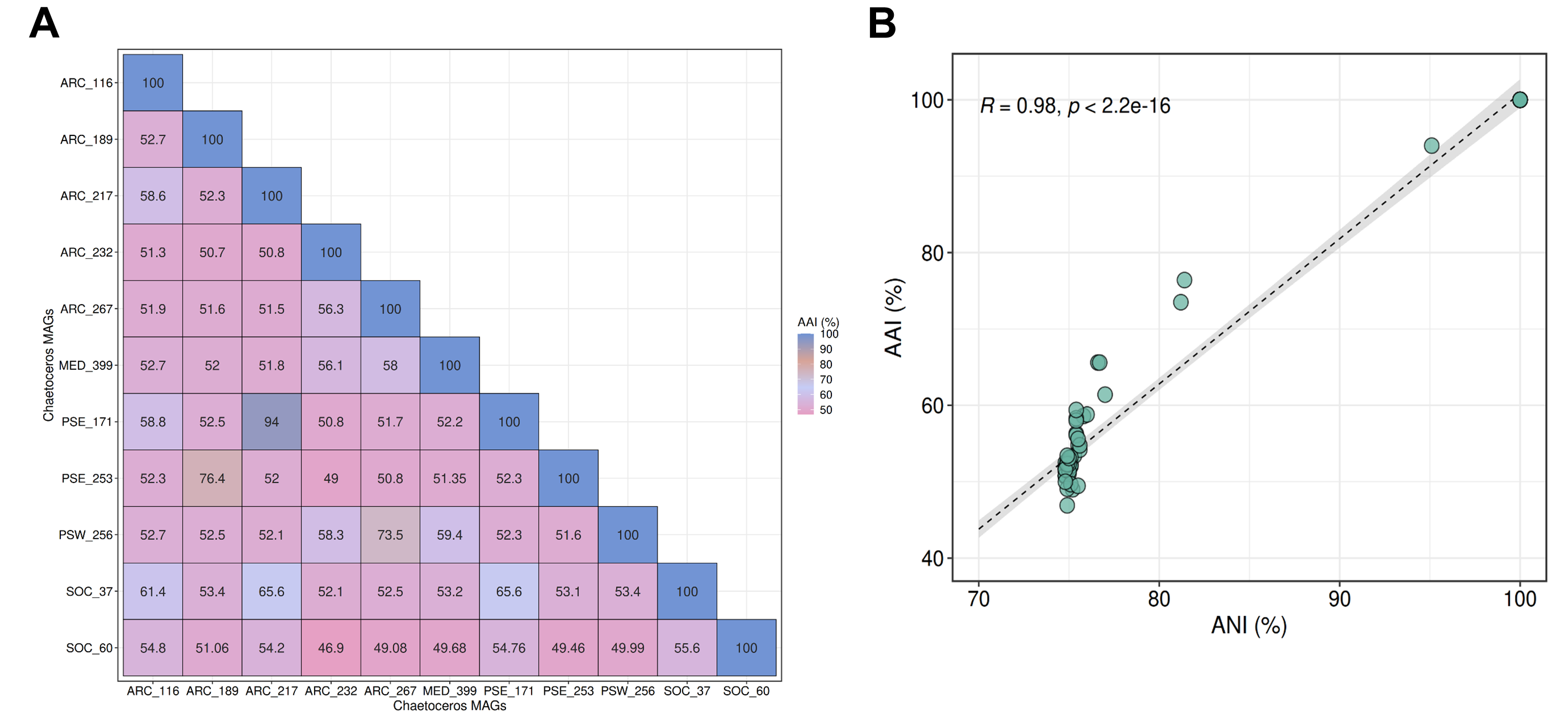

Supplement: S2 Fig — (A) AAI of the MAGs. (B) Correlation analysis between ANI and AAI showing significant positive correlation (Pearson’s correlation, the shaded area corresponds to 95% confidence interval). (PNG) [file pbio.3001893.s009.png]

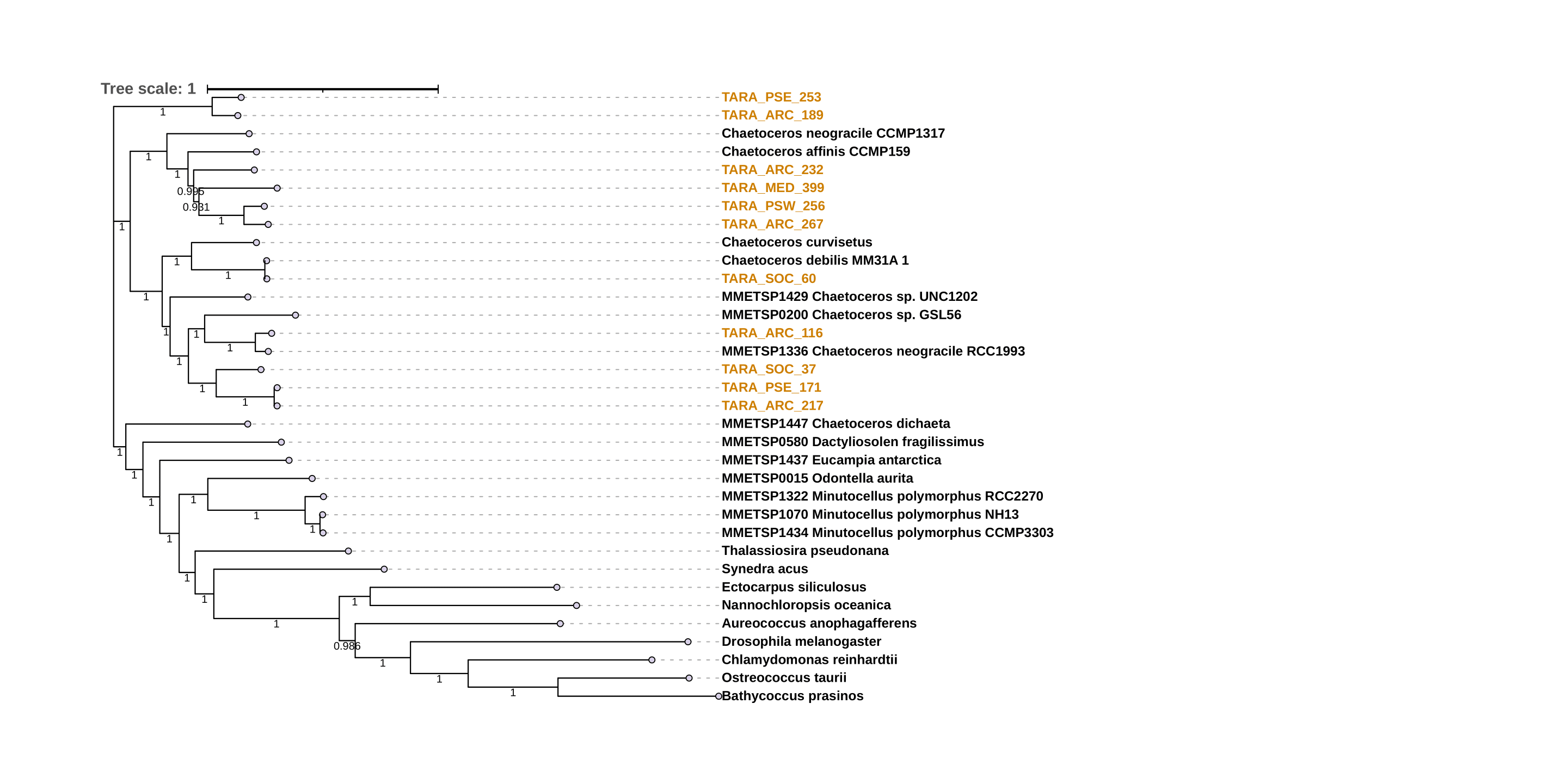

Supplement: S3 Fig — The tree was based on 846 orthogroups containing at least 50% of species having single-copy genes in any orthogroup. Support values are indicated on the branches. (PNG) [file pbio.3001893.s010.png]

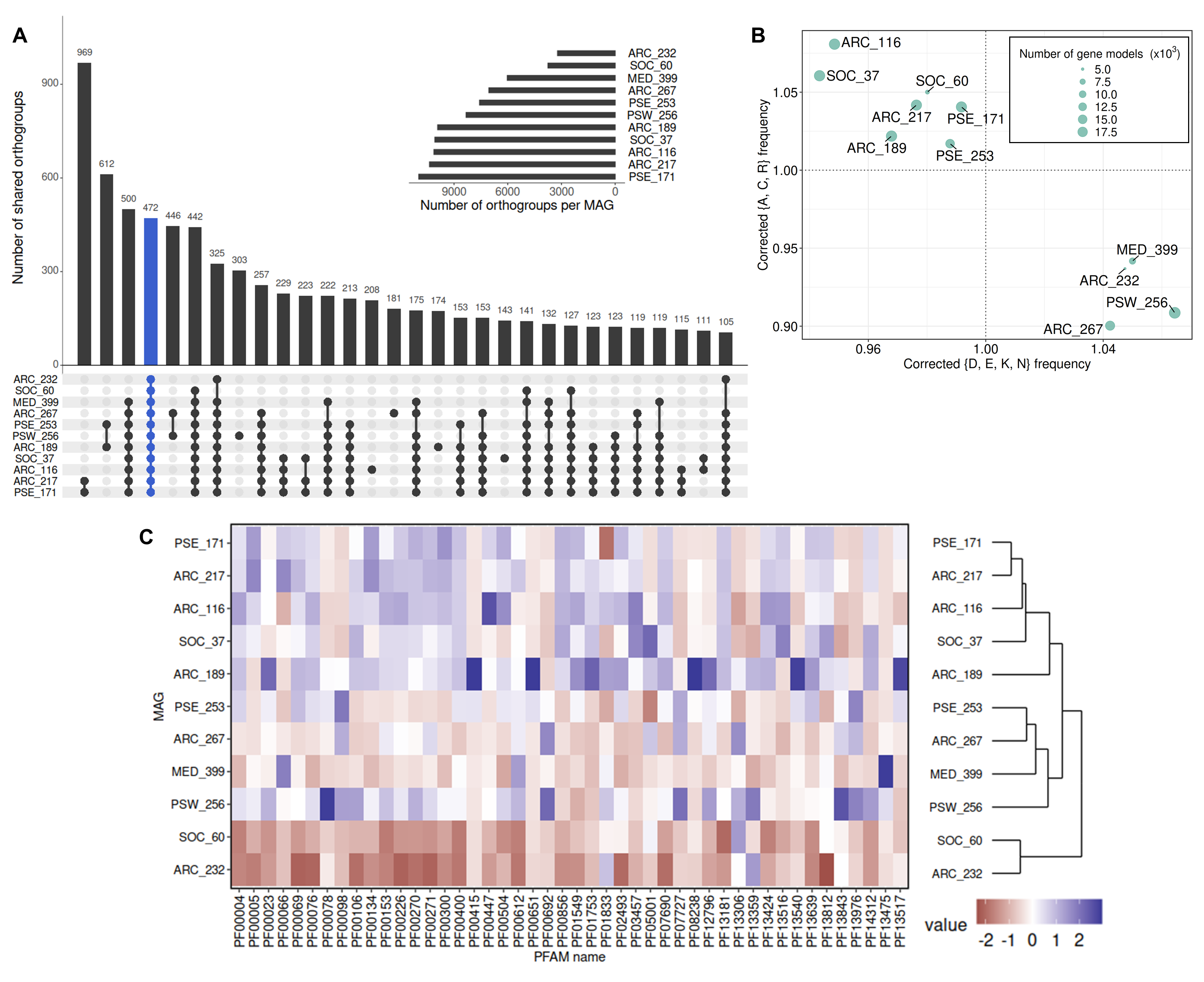

Supplement: S4 Fig — (A) Upset plot representing the top 30 shared orthogroups among the MAGs, with the orthogroups shared by all genomes highlighted in blue. (B) Frequency of the most variable amino acids compared to their global means across all MAGs. The MAG respective number of genes is indicated for comparison. (C) Heatmap of 46 PFAM domains displaying the most variable copy number (SD ≥ 10) among the MAGs (see S2 Table for details). (PNG) [file pbio.3001893.s011.png]

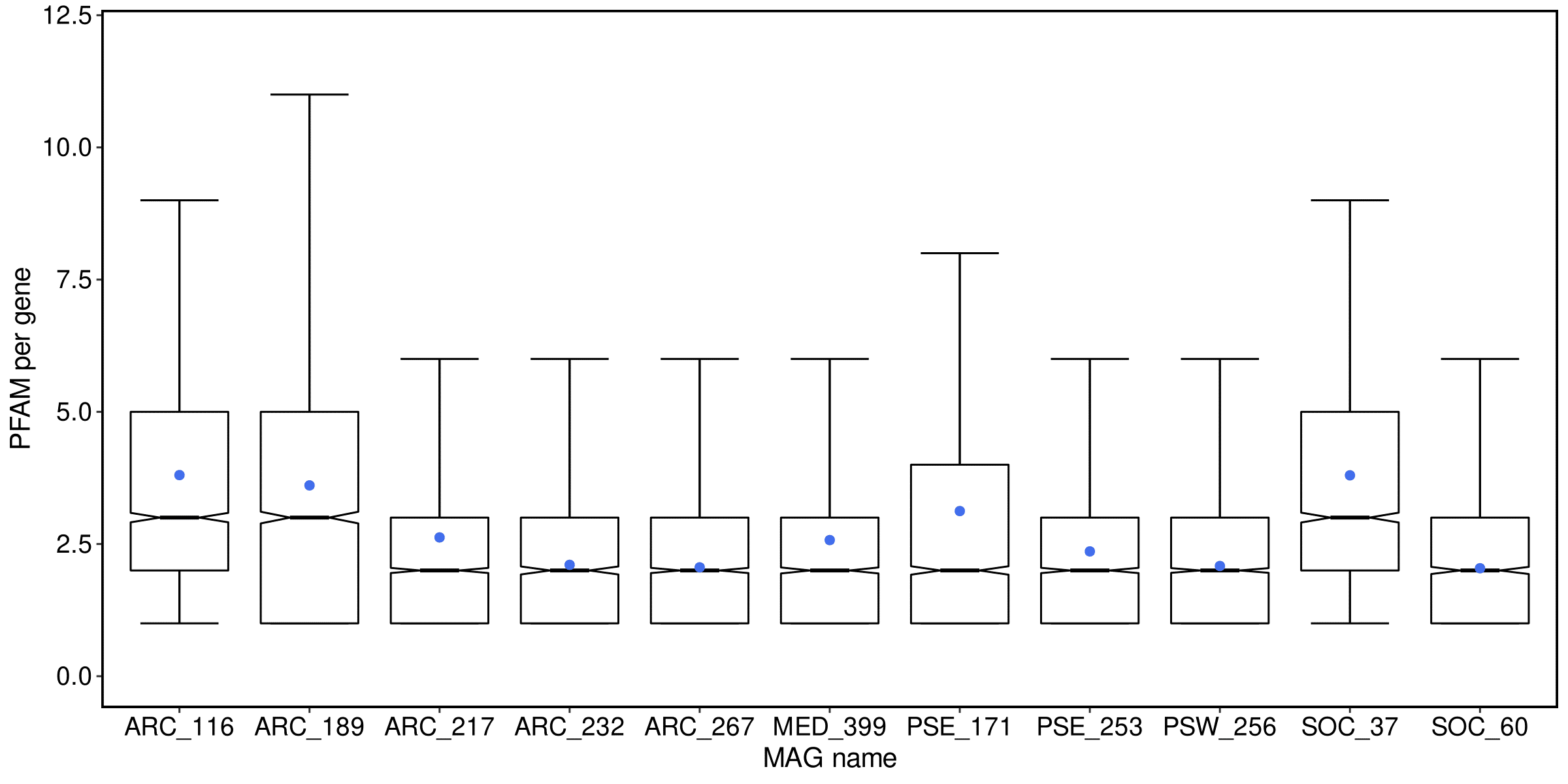

Supplement: S5 Fig — Boxplots plotting the distribution of the number of PFAM domains per gene with the blue dot representing the mean. (PNG) [file pbio.3001893.s012.png]

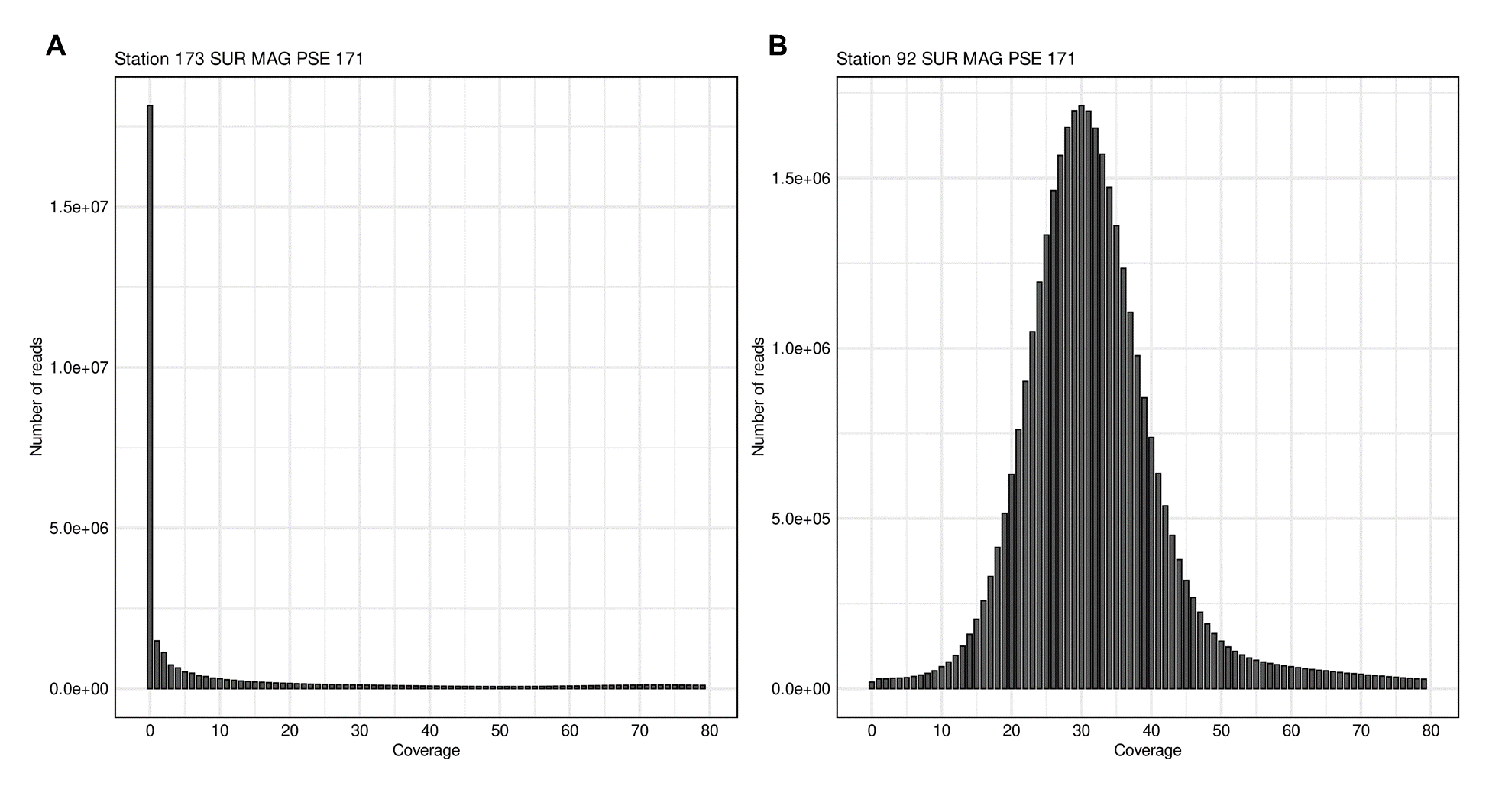

Supplement: S6 Fig — Read coverage distribution of MAG PSE_171 at the surface of stations (A) TARA_173 and (B) TARA_92. The distribution at station TARA_173 does not display enough coverage depth nor a clear unimodal pattern and will be discarded, whereas the pattern of TARA_92 is clearly unimodal and centered around 30×, hence the reads from this station will be kept for further analyses. (PNG) [file pbio.3001893.s013.png]

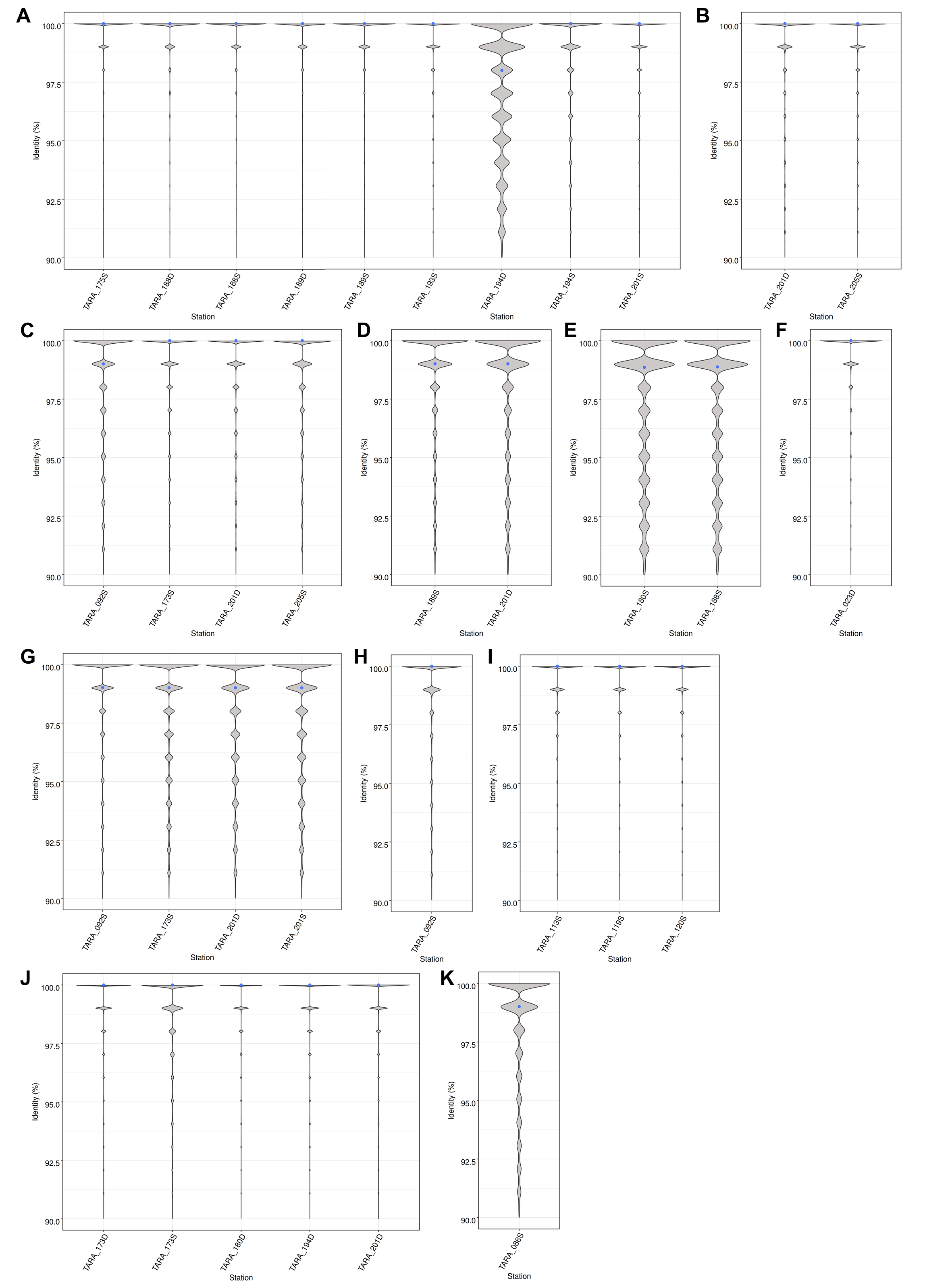

Supplement: S7 Fig — (A) ARC_116, (B) ARC_189, (C) ARC_217, (D) ARC_232, (E) ARC_267, (F) MED_399, (G) PSE_171, (H) PSE_253, (I) PSW_256, (J) SOC_37, and (K) SOC_60. The blue dots represent mean identity. (PNG) [file pbio.3001893.s014.png]

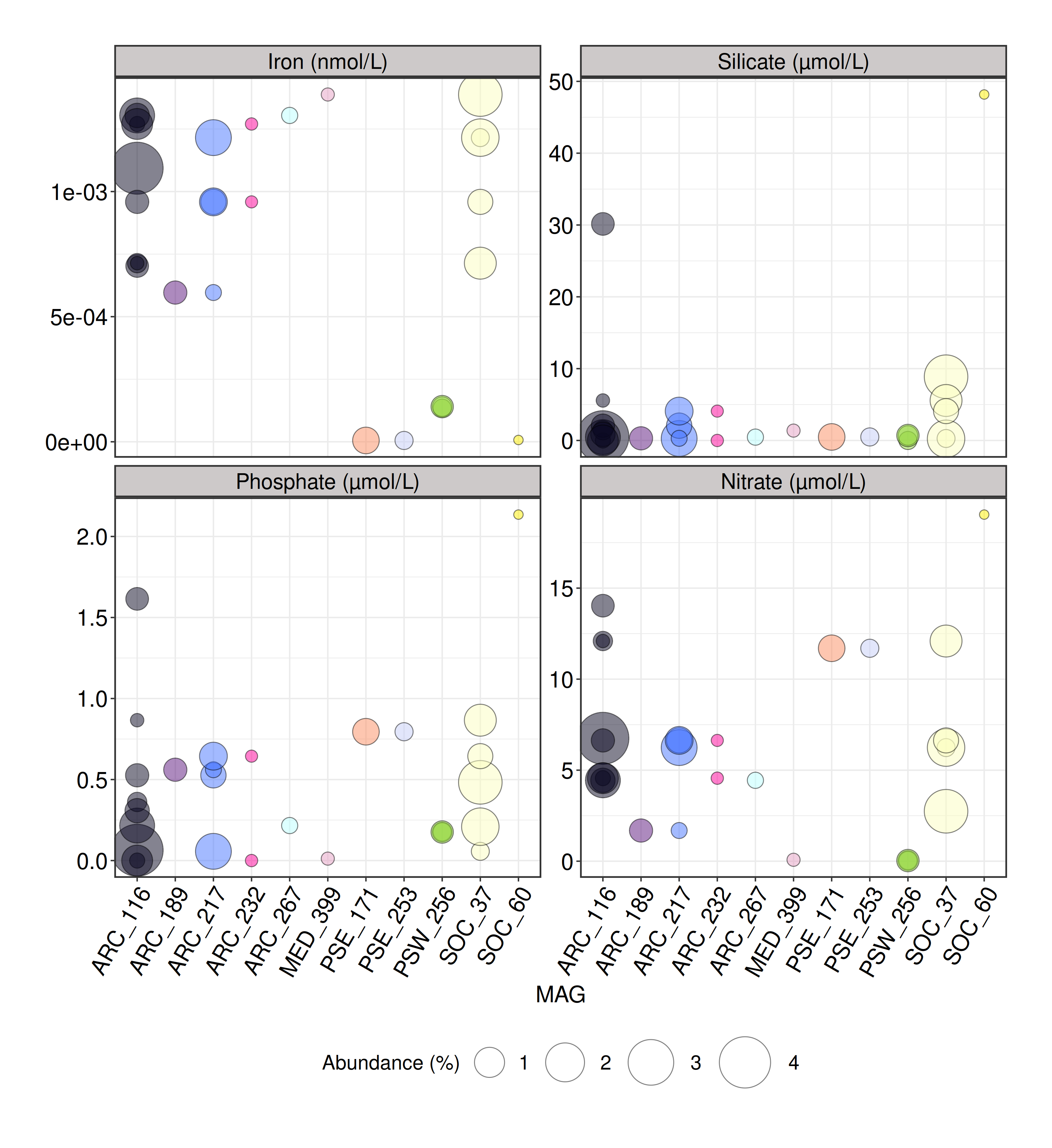

Supplement: S8 Fig — For a given MAG, most of the populations are distributed across a rather wide spectrum of iron, silicate, phosphate, and nitrate concentrations. (PNG) [file pbio.3001893.s015.png]

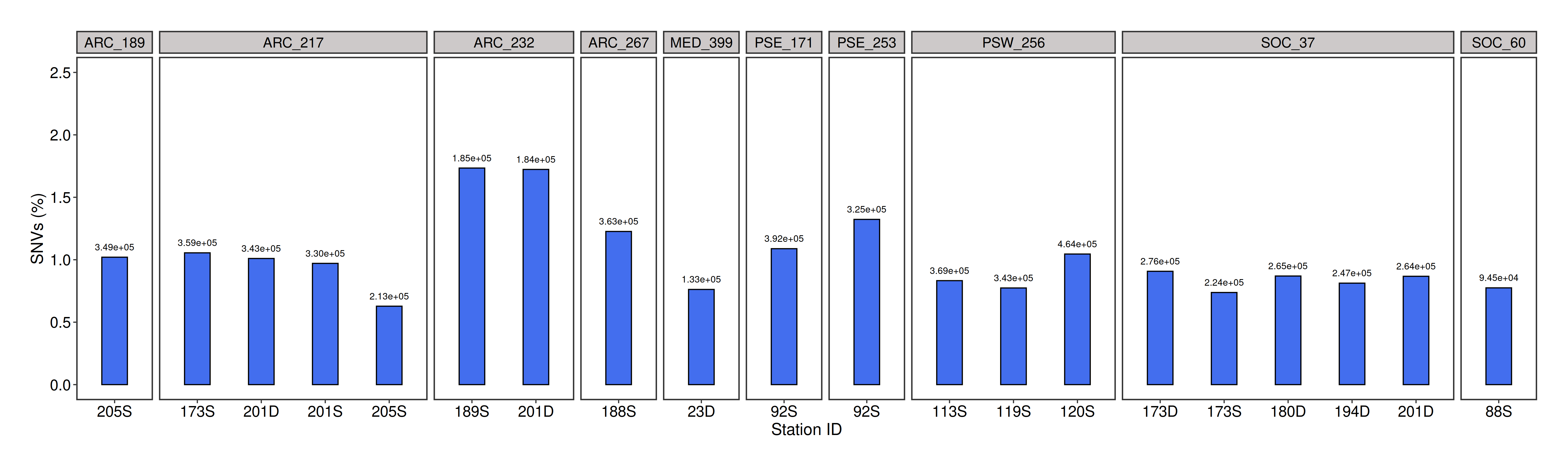

Supplement: S9 Fig — SNV percentages are shown, together with the total number of variants. (PNG) [file pbio.3001893.s016.png]

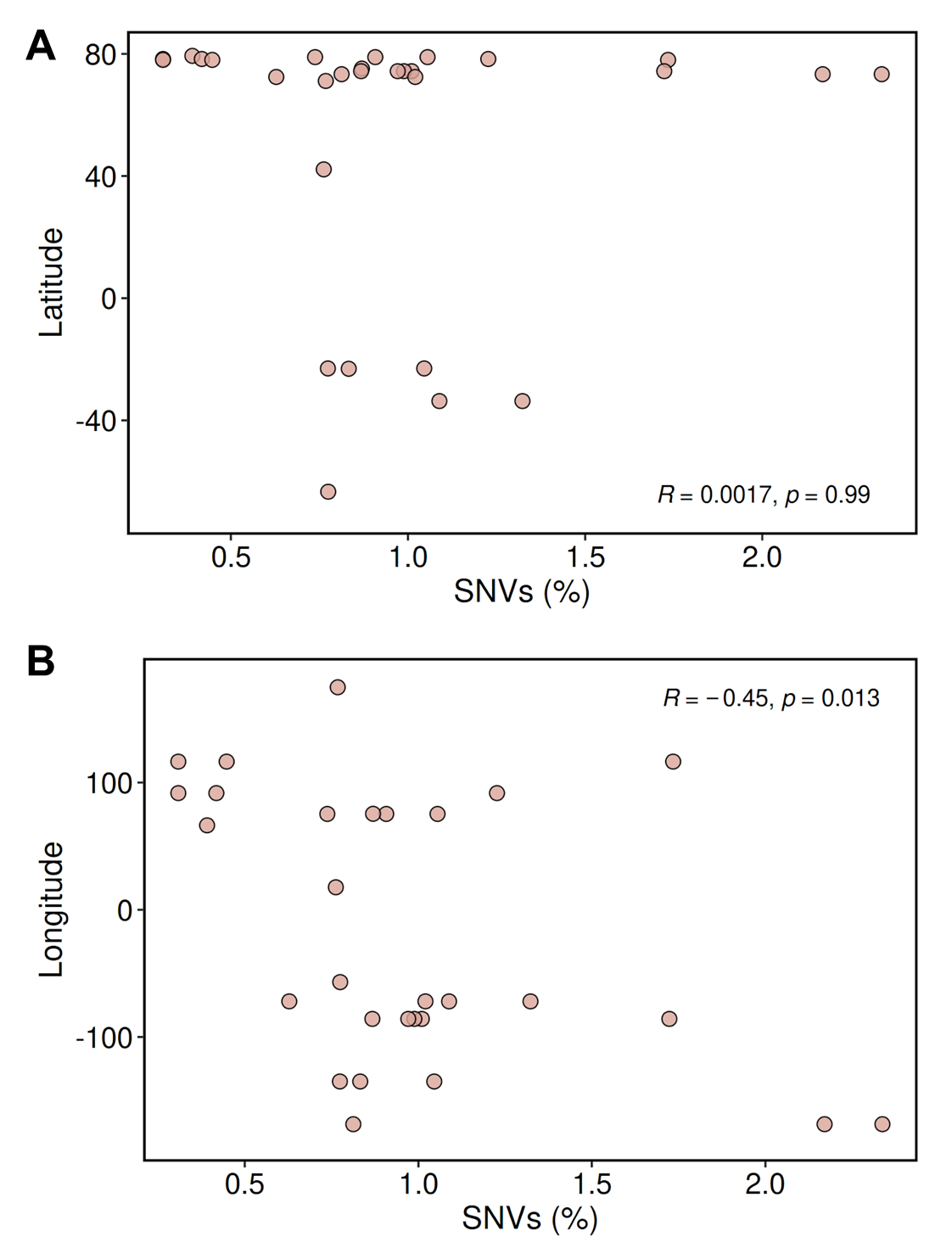

Supplement: S10 Fig — Scatterplots showing the repartition of the SNVs in the populations regarding their (A) latitude and (B) longitude (Pearson’s correlation coefficients and p-values are shown). No correlation was observed for the latitude while a negative effect of the longitude was observed. (PNG) [file pbio.3001893.s017.png]

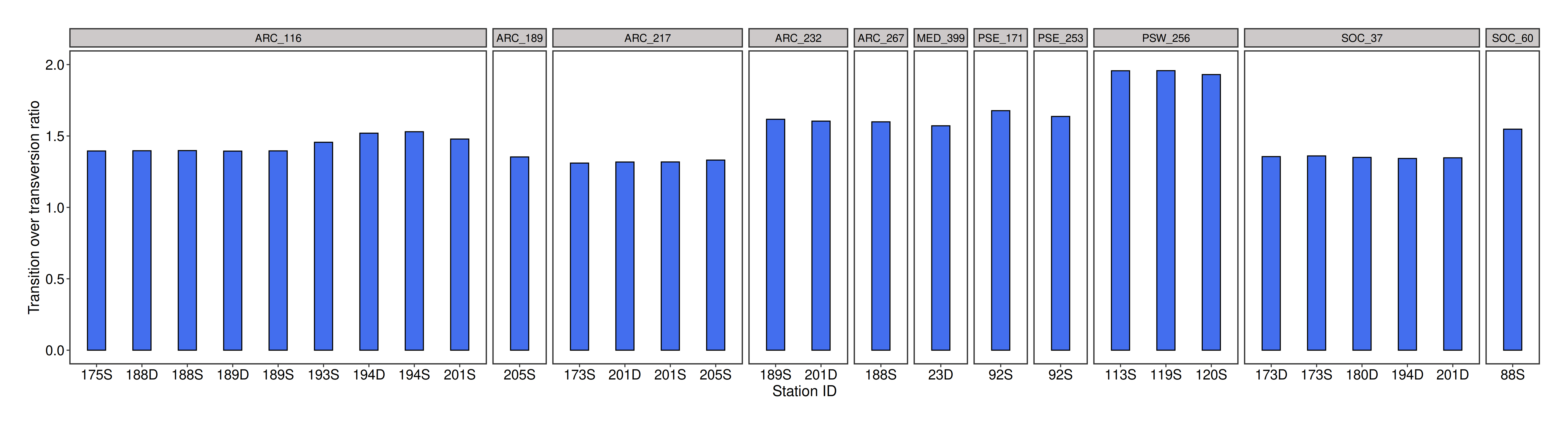

Supplement: S11 Fig — (PNG) [file pbio.3001893.s018.png]

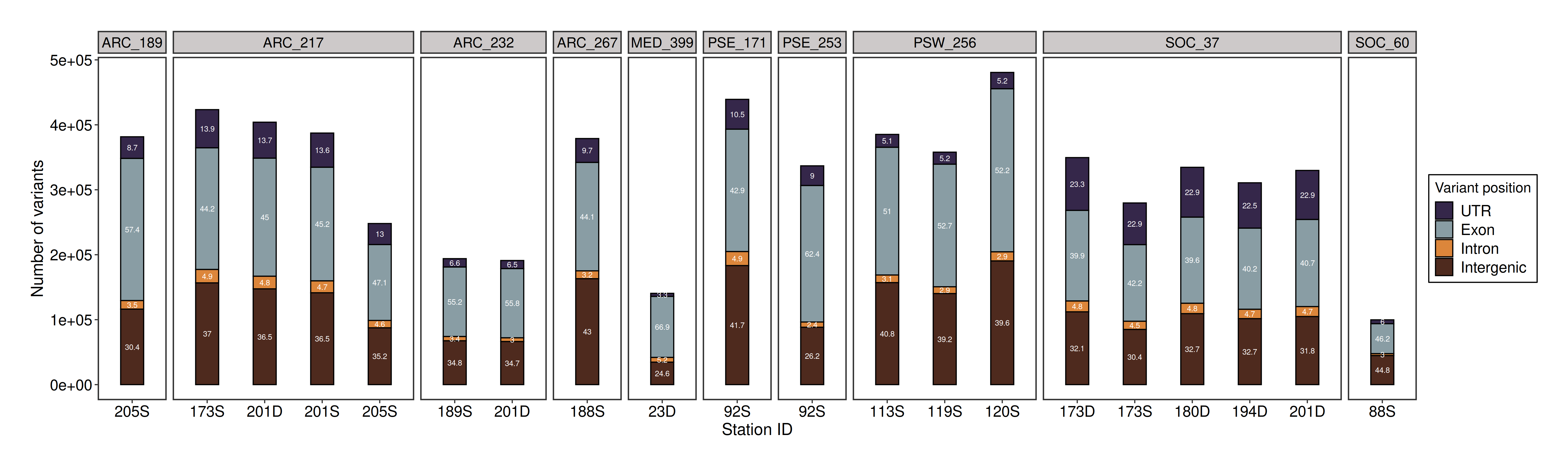

Supplement: S12 Fig — The exact proportion of each category is annotated. (PNG) [file pbio.3001893.s019.png]

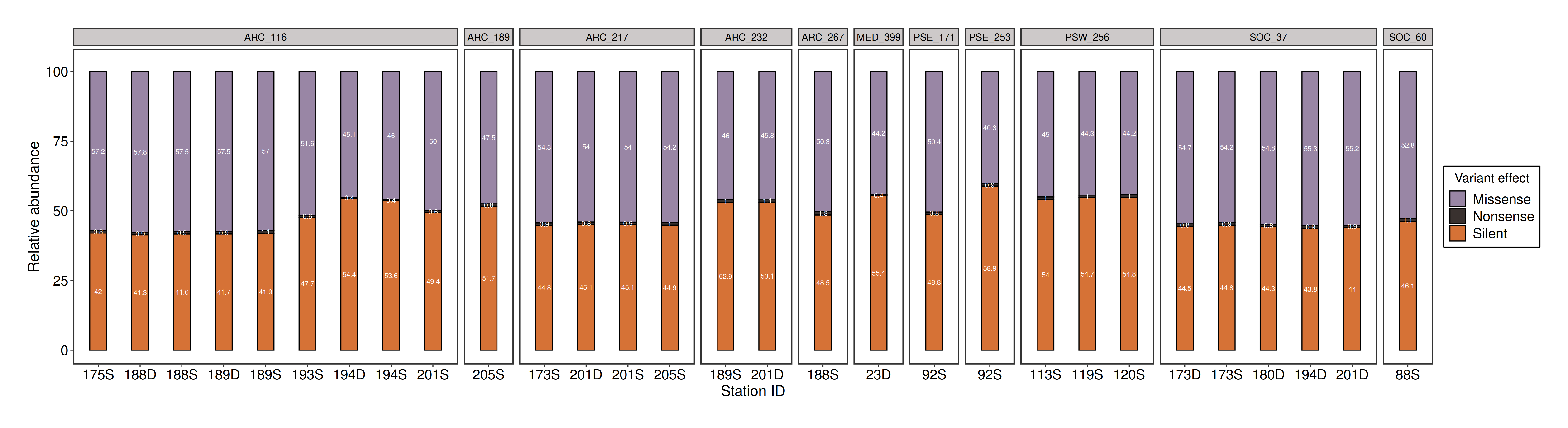

Supplement: S13 Fig — (PNG) [file pbio.3001893.s020.png]

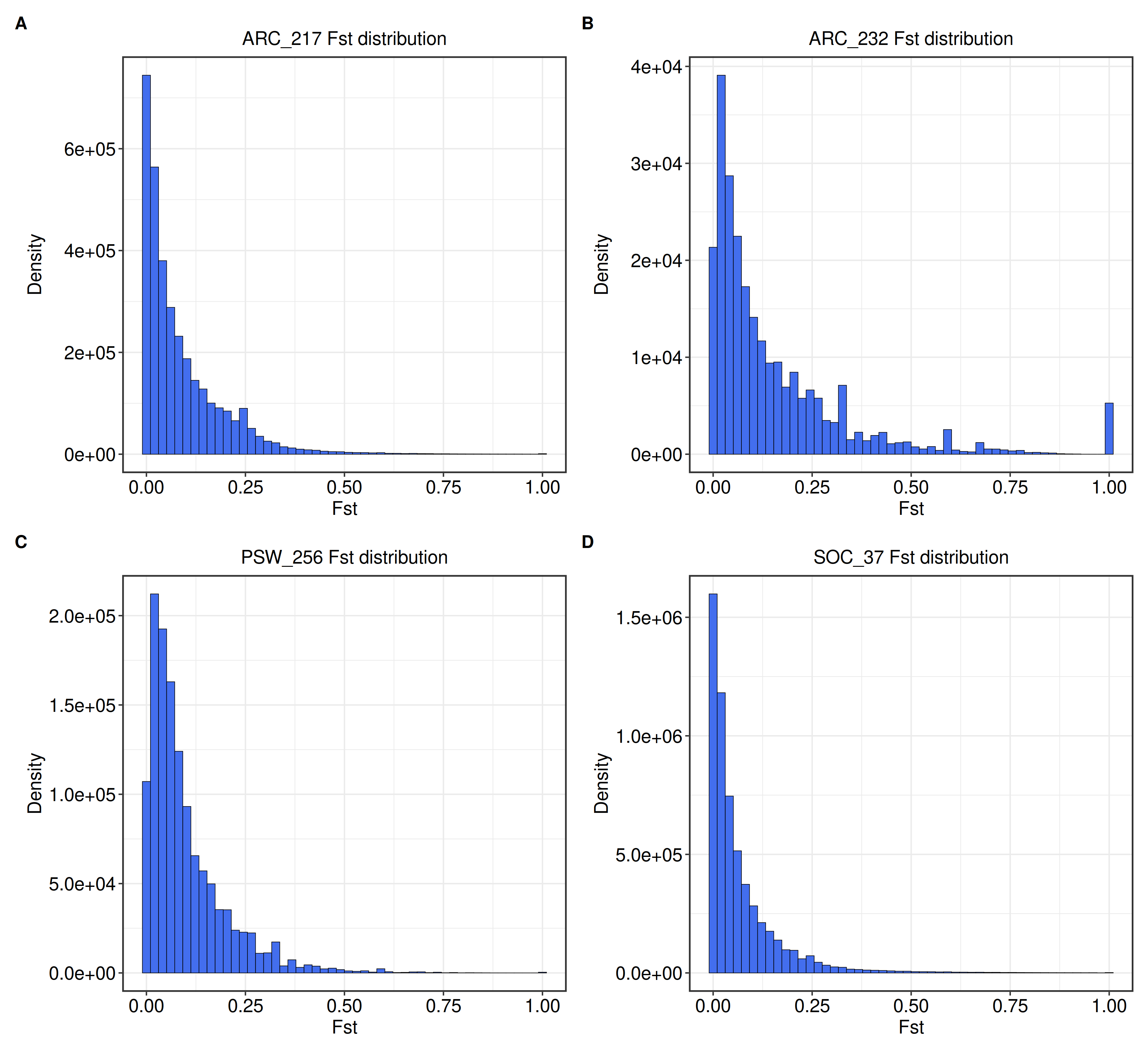

Supplement: S14 Fig — The distributions are shown for the MAGs presenting at least 2 populations, that is (A) ARC_217, (B) ARC_232, (C) PSW_256, and (D) SOC_37. The FST distributions appear globally unimodal, confirming that the reads of the respective MAGs were recruited from a single species. (PNG) [file pbio.3001893.s021.png]

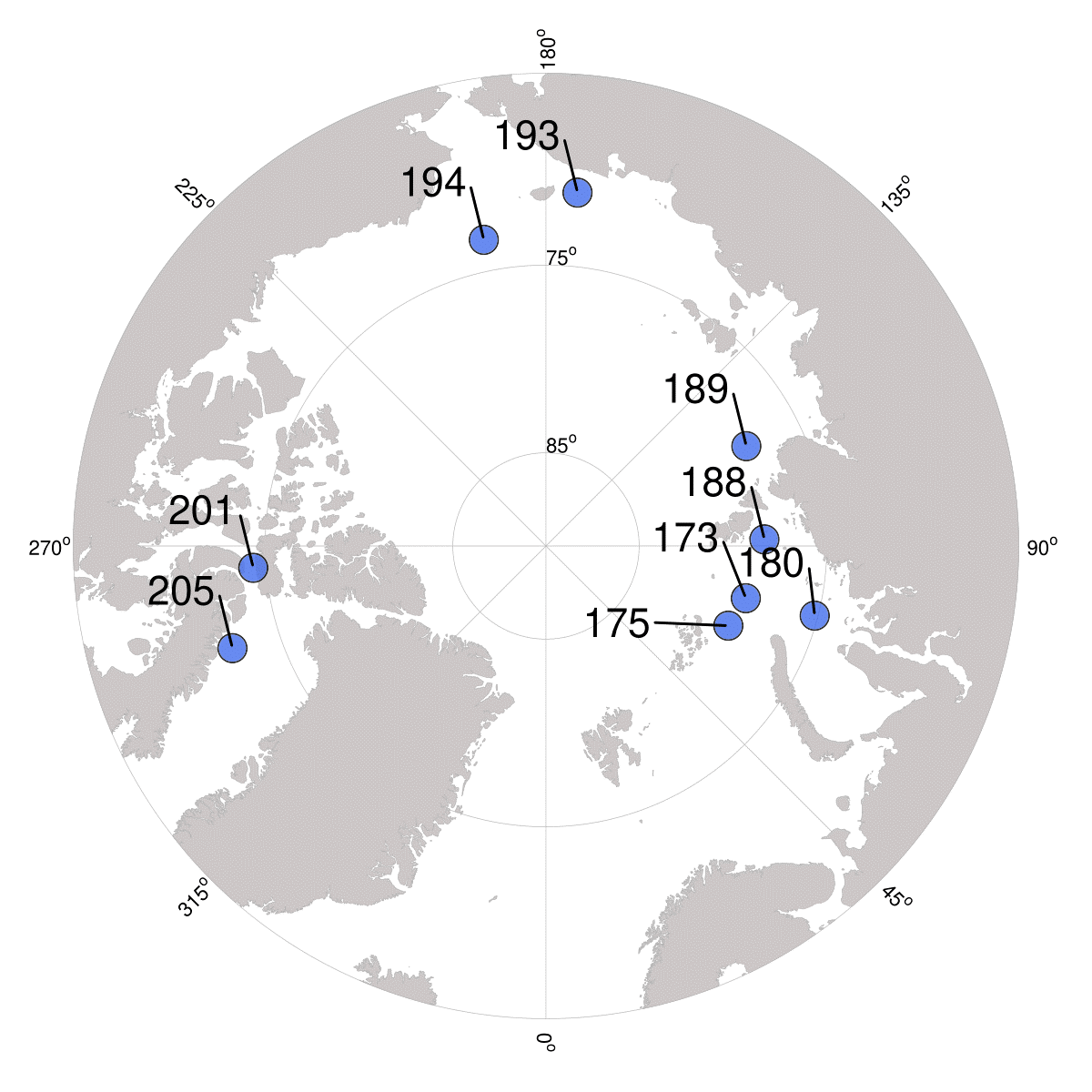

Supplement: S15 Fig — The Tara sampling stations belong to Arctic zones as follows: Atlantic-Arctic (175), Kara-Laptev (173, 180, 188, and 189), Pacific-Arctic (193 and 194), Arctic Archipelago (201), and Davis-Baffin (205) (based on Royo-Llonch and colleagues, 2021). The R packages “oce” v1.7–6 [141] and “PlotSvalbard” v0.9.2 (https://github.com/MikkoVihtakari/PlotSvalbard) were used to build the maps centered on the Arctic Ocean. (PNG) [file pbio.3001893.s022.png]

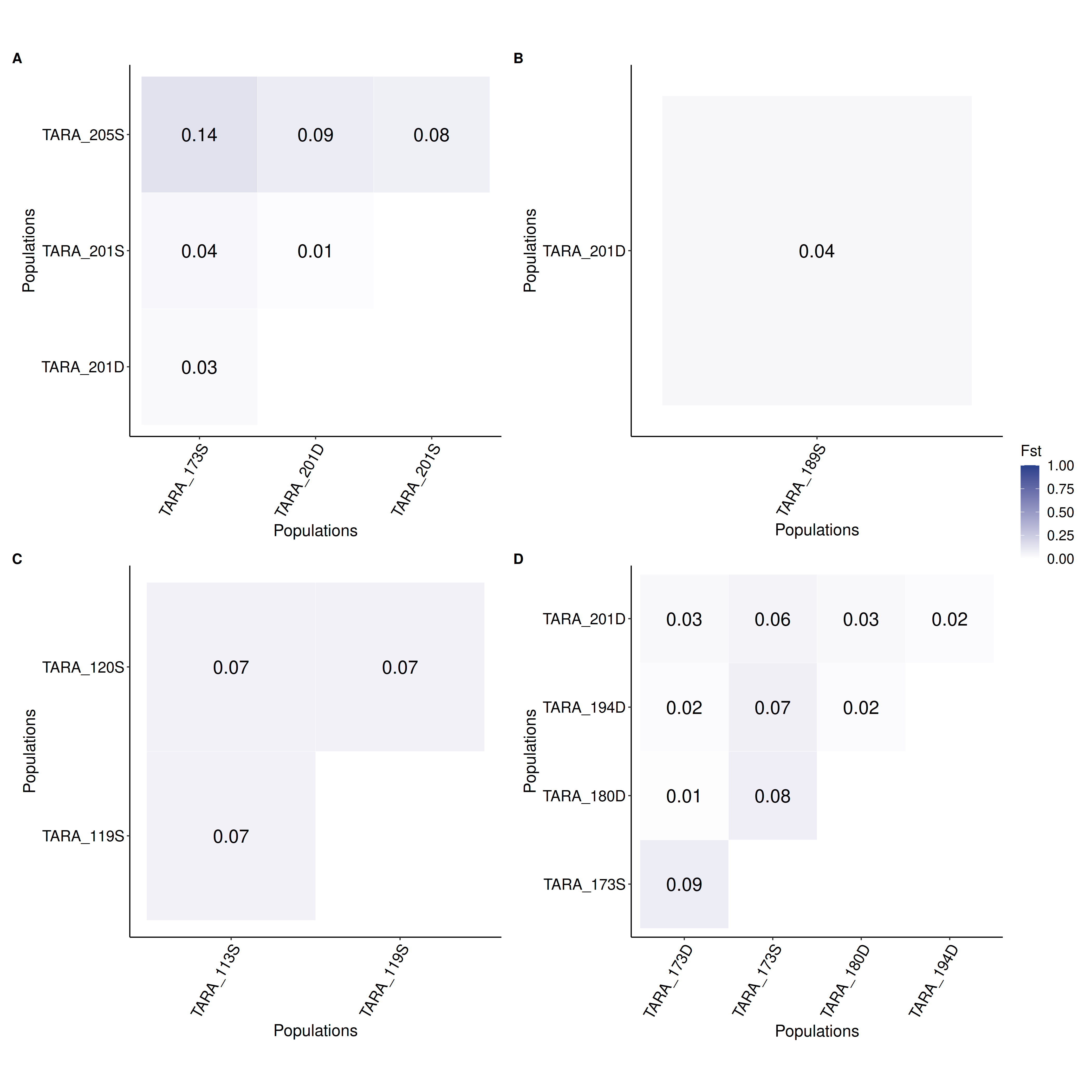

Supplement: S16 Fig — The matrices correspond to (A) ARC_217, (B) ARC_232, (C) PSW_256, and (D) SOC_37 populations. (PNG) [file pbio.3001893.s023.png]

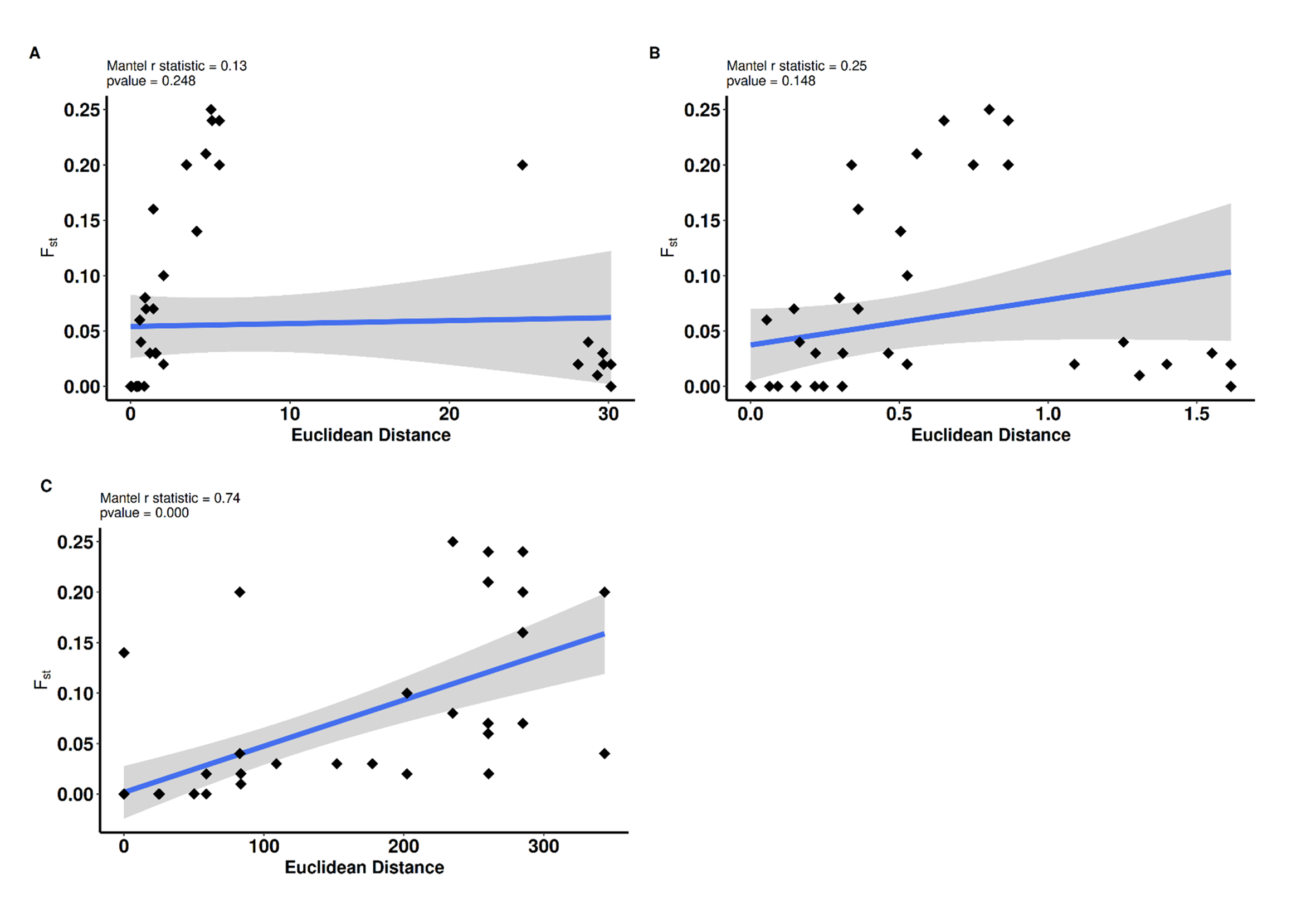

Supplement: S17 Fig — For (A) silicate, (B) phosphate, and (C) geographic distance. (PNG) [file pbio.3001893.s024.png]

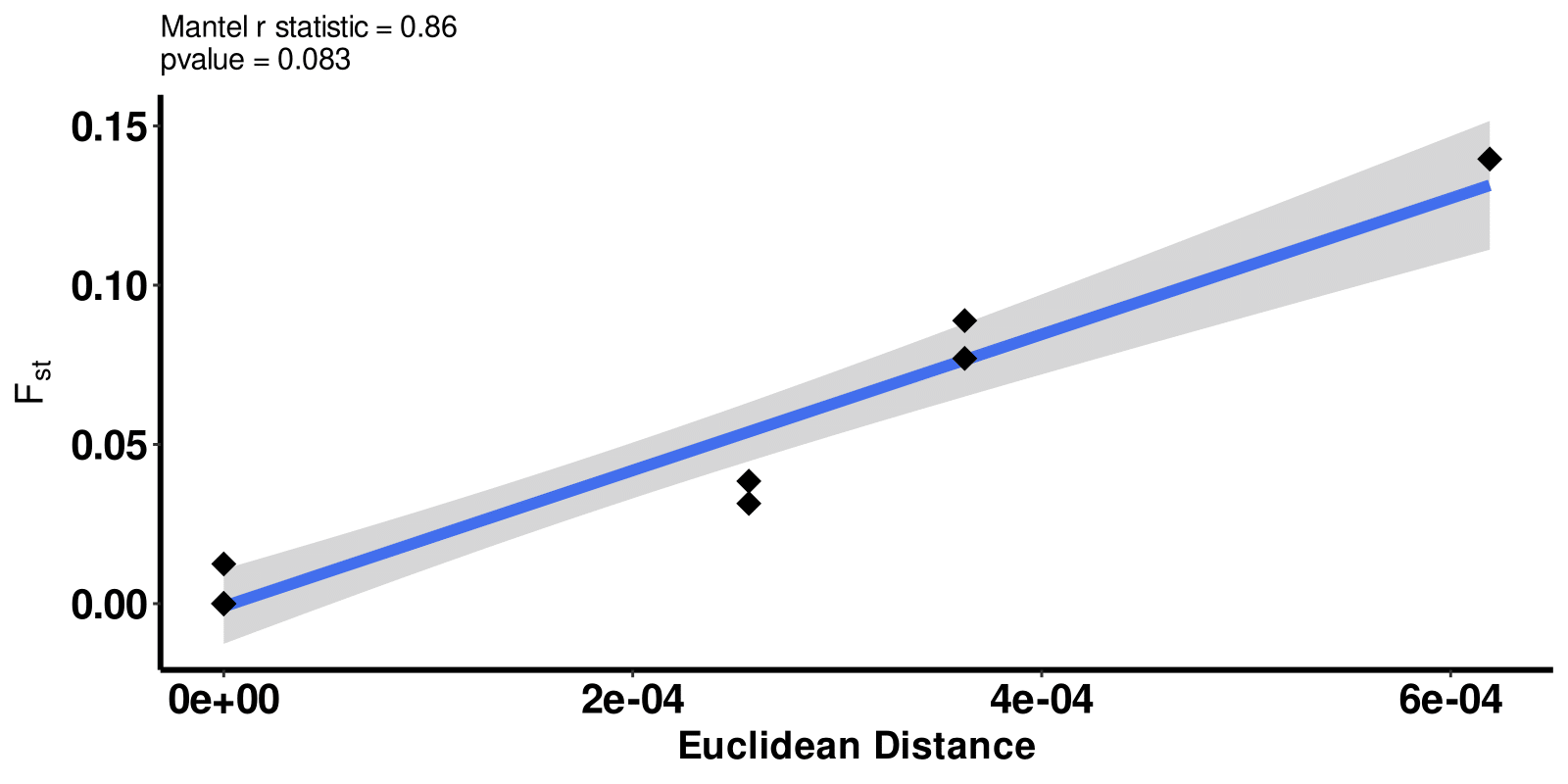

Supplement: S18 Fig — Shown for iron. (PNG) [file pbio.3001893.s025.png]

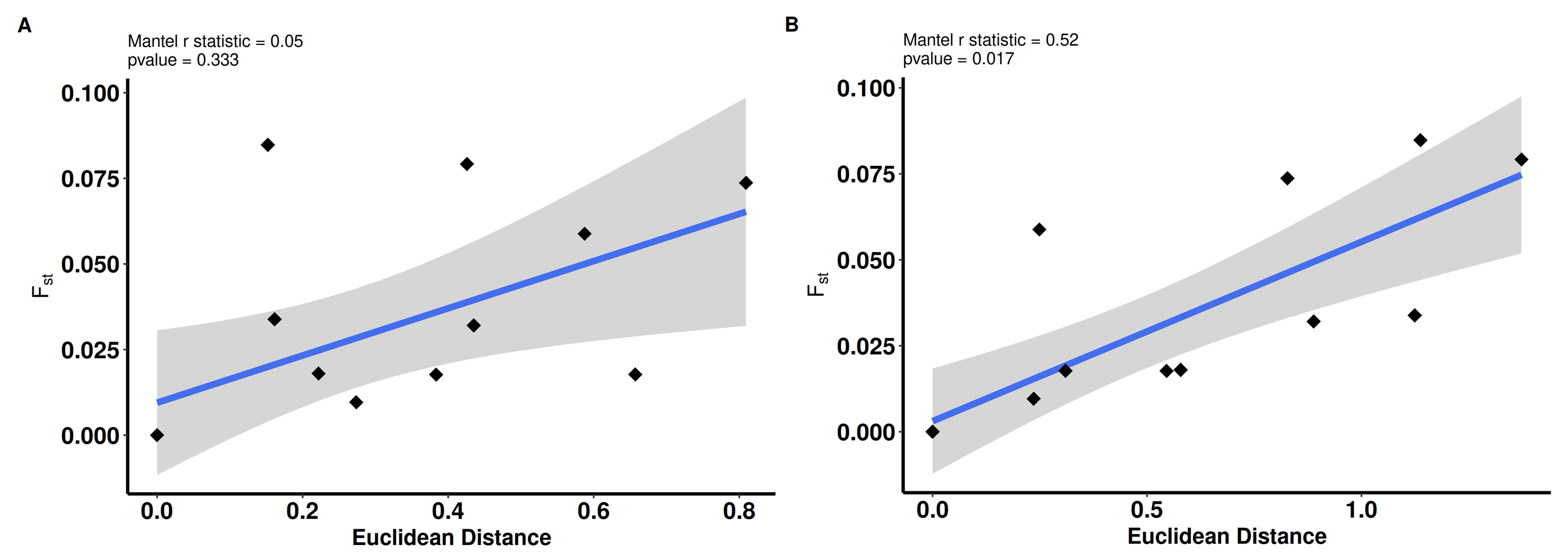

Supplement: S19 Fig — For (A) phosphate and (B) temperature. (PNG) [file pbio.3001893.s026.png]

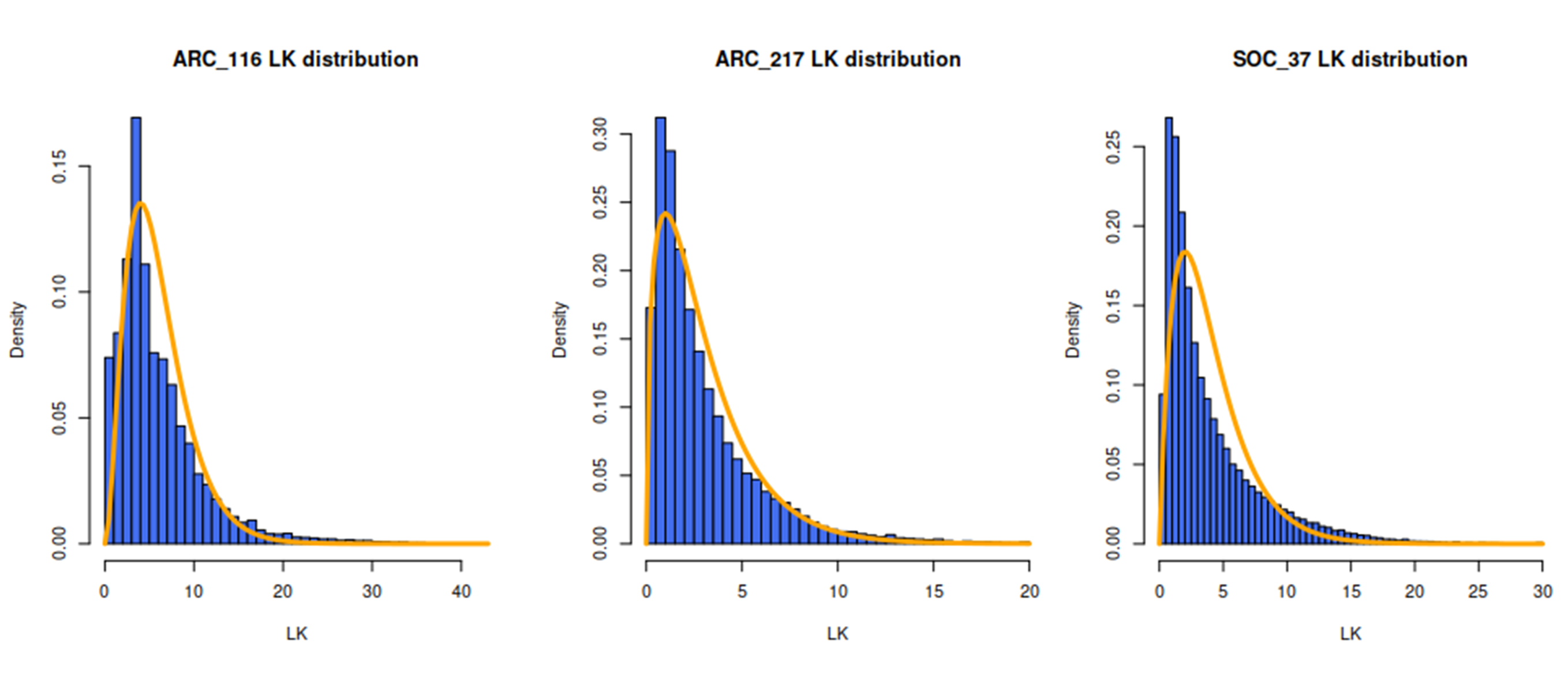

Supplement: S20 Fig — The orange line indicates the χ2 (df = 6; 3 and 4) theoretical distribution. The observed LK distributions followed the expected one. (PNG) [file pbio.3001893.s027.png]

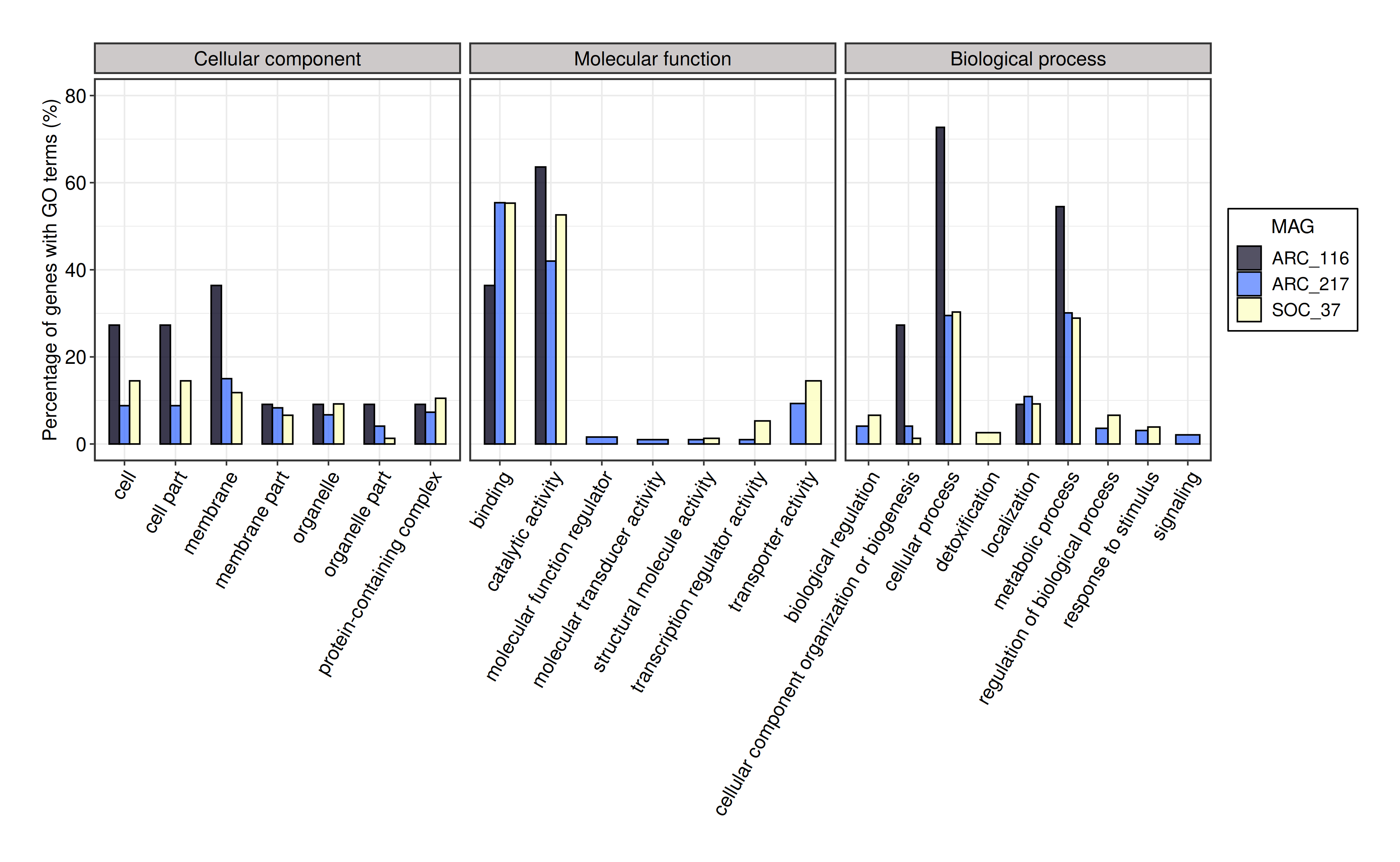

Supplement: S21 Fig — (PNG) [file pbio.3001893.s028.png]

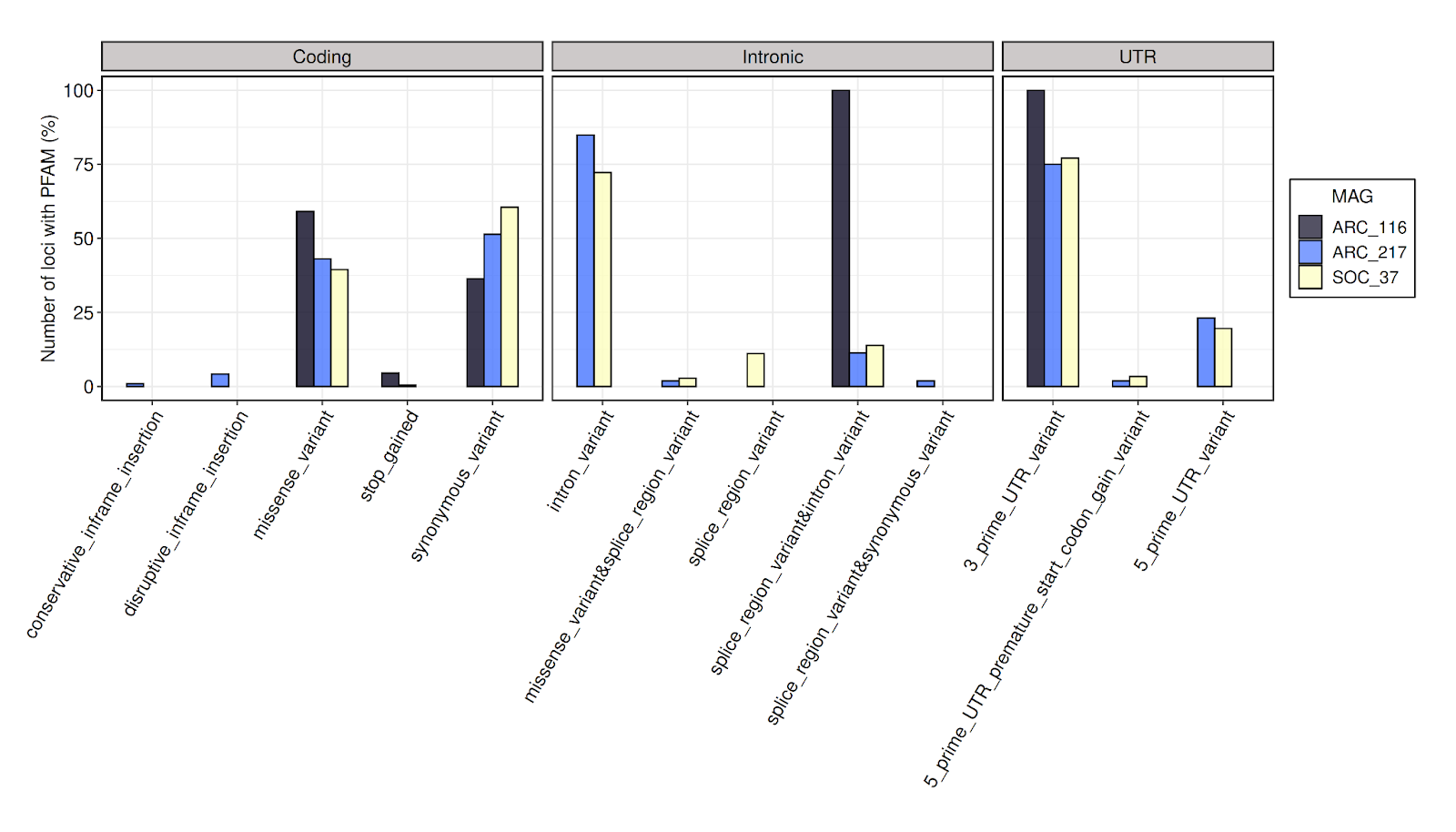

Supplement: S22 Fig — (PNG) [file pbio.3001893.s029.png]

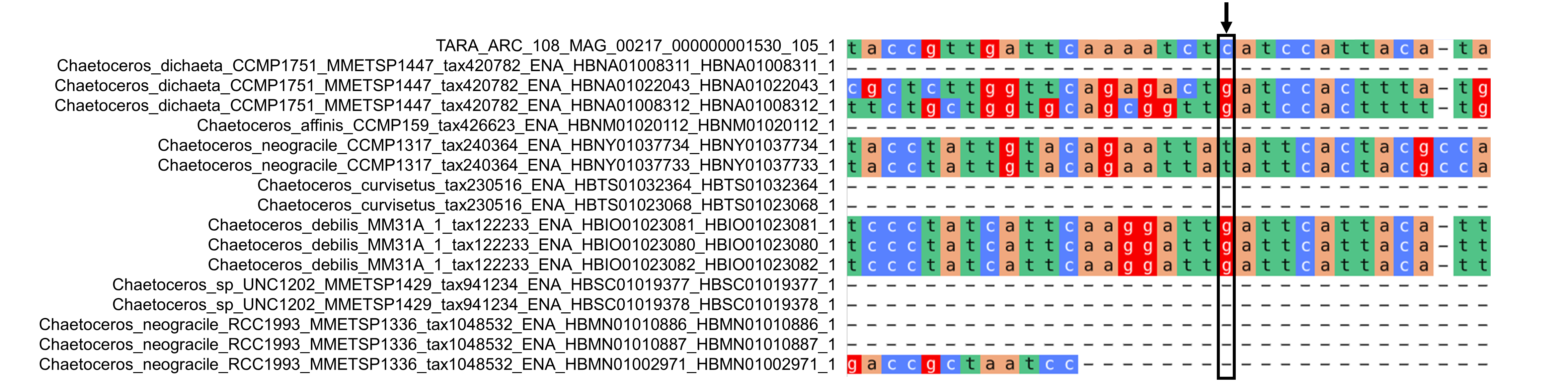

Supplement: S23 Fig — Sequence alignment was realised with MAFFT v7 with the gene 1530.105.1 set as reference sequence. The position of the reference nucleotide undergoing selection in some ARC_217 populations is highlighted. The sequences and alignment are in S1 and S2 Data files. (PNG) [file pbio.3001893.s030.png]

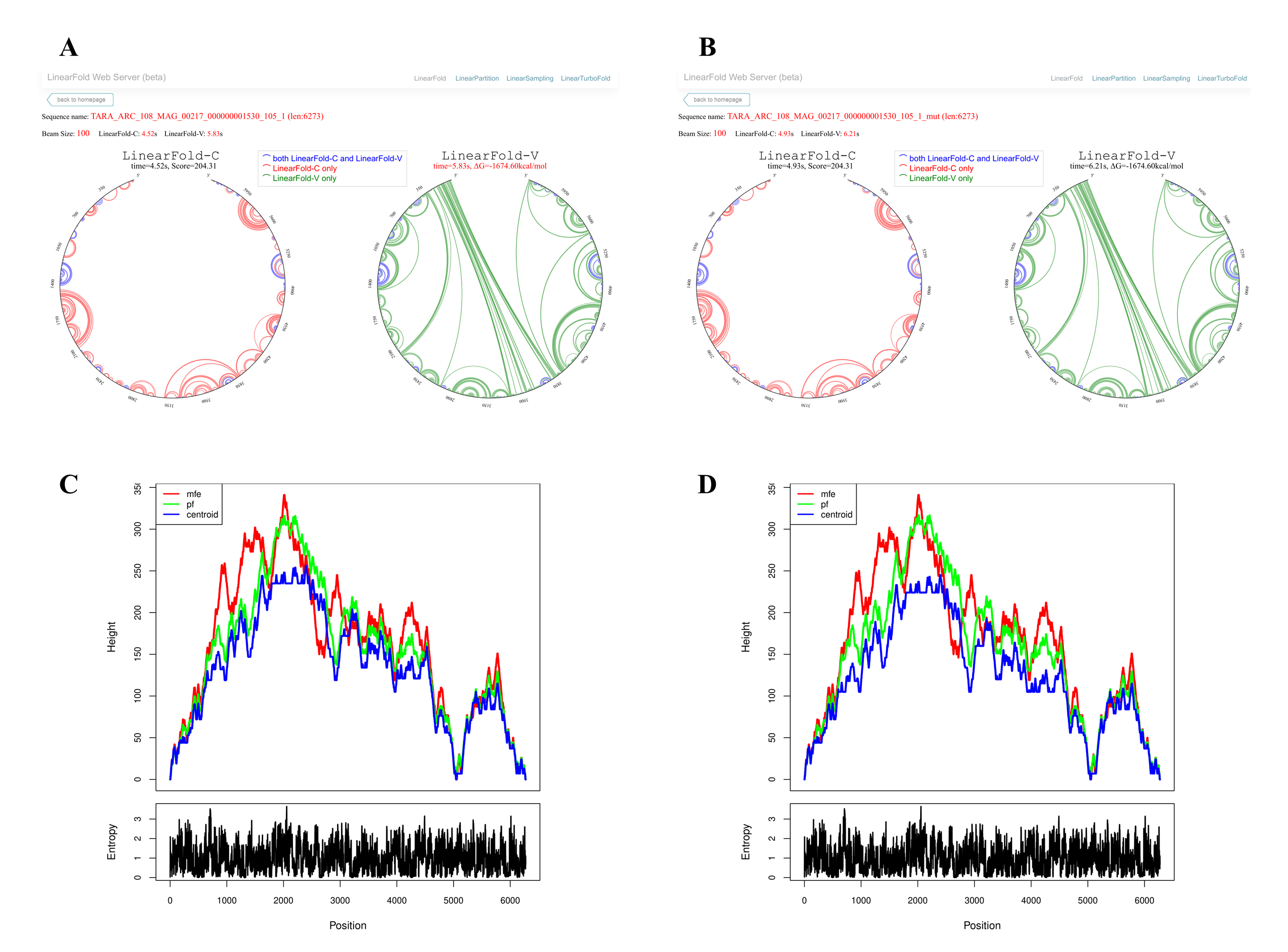

Supplement: S24 Fig — Outputs from the LinearFold webserver for (A) the reference gene and (B) the gene with the p.Leu277Leu mutation showing exactly the same structure patterns. Mountain plots and positional entropy outputs from RNAfold representing the minimum free energy structure for the (C) reference gene and (D) gene with the SNV, displaying slightly different patterns. (PNG) [file pbio.3001893.s031.png]

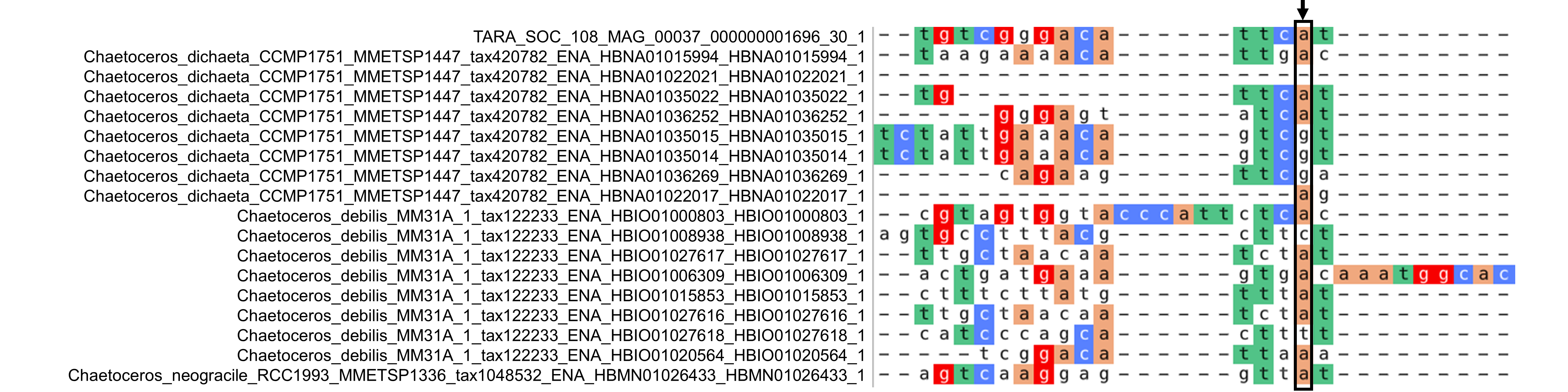

Supplement: S25 Fig — Sequence alignment was realised with MAFFT v7 with the gene 1696.30.1 set as reference sequence. The position of the reference nucleotide undergoing selection in some SOC_37 populations is highlighted. The sequences and alignment are in S3 and S4 Data files. (PNG) [file pbio.3001893.s032.png]

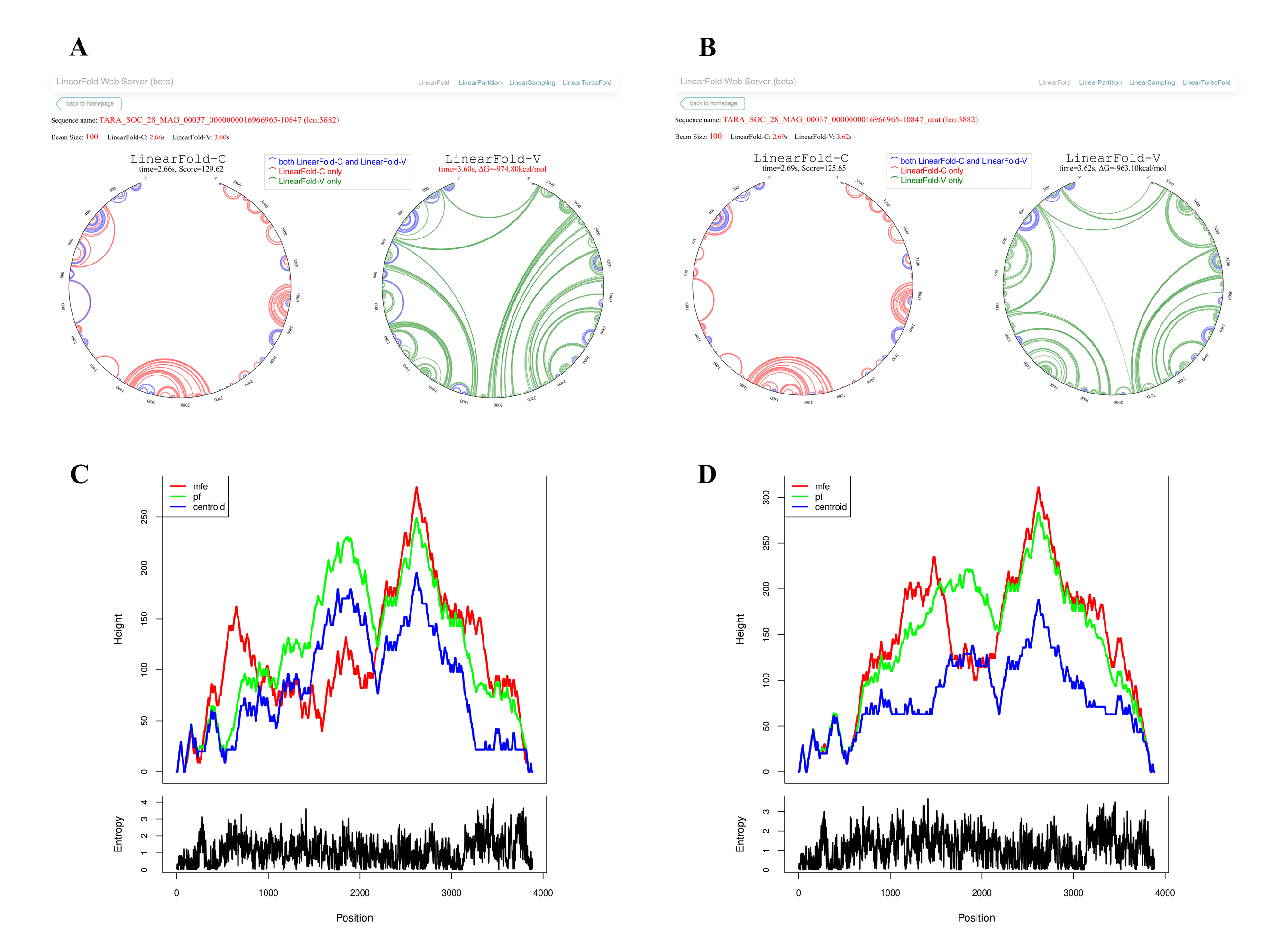

Supplement: S26 Fig — Outputs from the LinearFold webserver for (A) the reference gene and (B) the gene with the p.Met1063Leu mutation showing significantly different structure patterns. Mountain plots and positional entropy outputs from RNAfold representing the minimum free energy structure for the (C) reference gene and (D) gene with the SNV, displaying again different patterns. (PNG) [file pbio.3001893.s033.png]

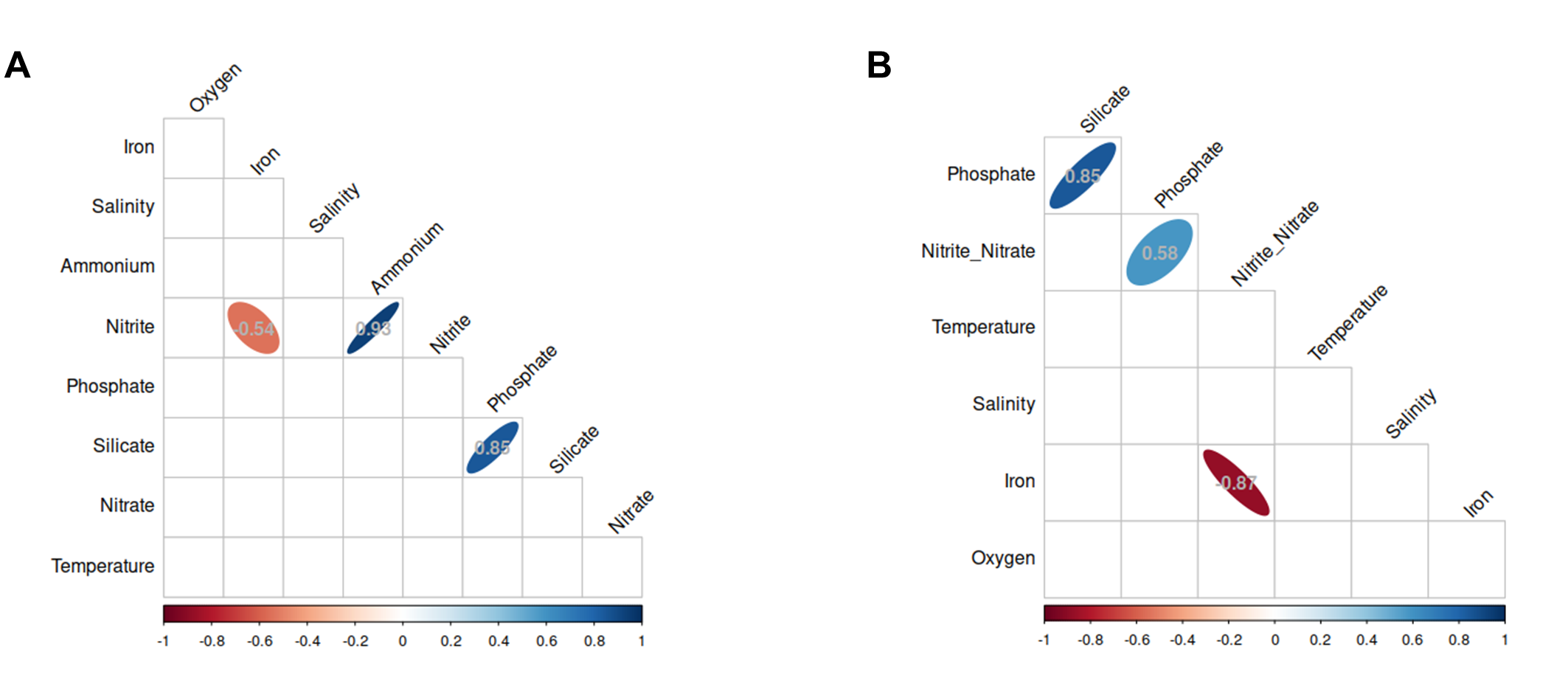

Supplement: S27 Fig — (A) Correlation matrix before simplification of the number of variables; (B) correlation matrix after variable simplification (values represent Spearman’s correlation rho, data are shown for significant correlations with p-value < 0.01). (PNG) [file pbio.3001893.s034.png]

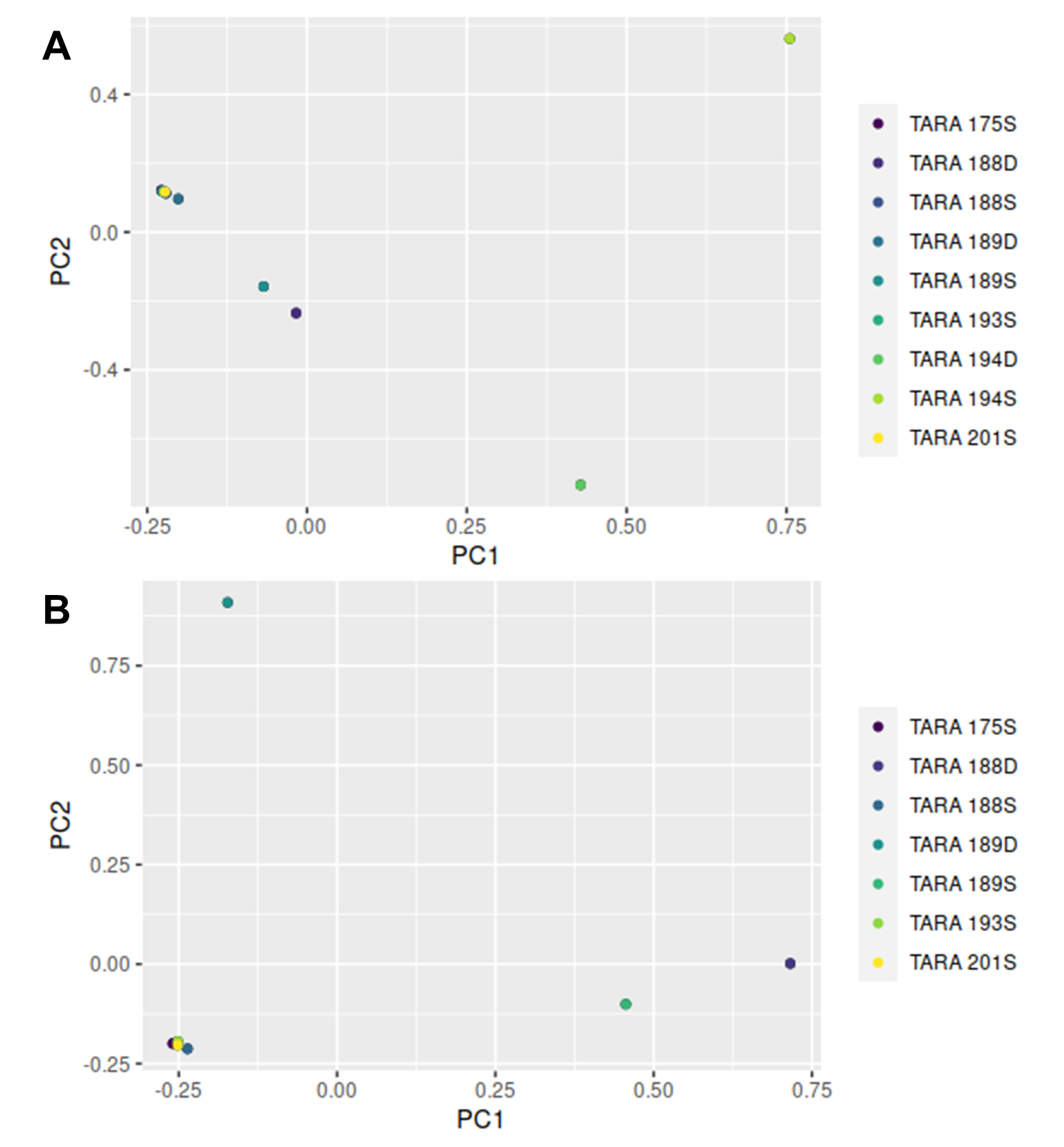

Supplement: S28 Fig — (A) The PCA of all the stations shows that populations from TARA_194 at the surface and DCM are pulled away from the others. (B) The PCA after removing the outlier points from station TARA_194 shows more details on the allelic frequency patterns, with less heterogeneous dispersion. Stations TARA_188D, TARA_189S and TARA_189D are clearly apart from the others. (PNG) [file pbio.3001893.s035.png]
