# Supplementary material for: Whole-genome scanning reveals environmental selection mechanisms that shape diversity in populations of the epipelagic diatom Chaetoceros
Source: PLoS Biol. 2022 Nov 28;20(11):e3001893. doi: 10.1371/journal.pbio.3001893 (PMC9731442; doi:10.1371/journal.pbio.3001893)
Supplement: S1 Text — (DOCX) [file pbio.3001893.s036.docx]

**Supporting information for the manuscript: Whole-genome scanning reveals environmental selection mechanisms that shape diversity in populations of the epipelagic diatom *Chaetoceros***

Nef et al. PLOS Biology

**Comparative analyses regarding orthogroups, amino acid content and PFAM domains**

Identifying the orthologous genes shared between the MAGs showed that the most elevated number of orthogroups were not shared by the 11 genomes, as one would have expected, but rather by the two MAGs ARC_217 and PSE_171 (969 orthogroups) (S4A Fig; Sheet A in S5 Data). The second highest set of orthogroups was shared between ARC_189 and PSE_253 (612 orthogroups). The third group consisted of all the genomes except ARC_232 and SOC_60 (500 orthogroups), suggesting that the two excluded MAGs are the most divergent. This pattern is consistent with the result of the multigene phylogeny (see Fig 3B), and may be partly explained by the fact that both these genomes are the smallest in size, number of genes and BUSCO completion (see Fig 2A-C). Another explanation would be an artefactual result due to low genome completion. A total of 472 orthogroups appeared common to the 11 genomes. MAG-specific orthogroup sets were retrieved, with PSW_256 displaying the highest number of MAG-specific orthogroups (303), which may be because this genome is the largest both in size and gene number.

Two distinct genome groups were identified based on their predicted amino acid content. A first group, composed of ARC_116-217 and all genomes associated to PSE and SOC, exhibited an enrichment in arginine (R) as charged residues, in alanine (A) for the hydrophobic ones as well as in cysteine (C); the other group, consisting of ARC_232 and 267, MED_399 and PSW_256, formed a monophyletic group, and showed a larger proportion of aspartate (D), glutamate (E) and lysine (K) as charged residues, and in asparagine (N) (S4B Fig; Sheet B in S5 Data). Such differences in amino acid composition of predicted proteomes have been previously identified in a study investigating more than 100 algal genomes, in which saltwater algae encoded higher proportions of D, E and K residues and lower proportions of A and C compared to freshwater species [1].

Most MAGs showed a median of two PFAM domains per gene, with ARC_116, ARC_189 and SOC_37 among the most complete MAGs, displaying 3 domains per gene (S5 Fig; Sheet C in S5 Data). A selection of the most variable PFAM domains among the MAGs was conducted on those displaying a standard deviation at least equal or superior to 10, leading to the identification of 46 PFAMs (see S3 Table for detailed information) whose relative enrichments are represented on a heatmap (S4C Fig). The group formed by ARC_232 and SOC_60 displayed globally the same patterns of PFAM enrichment and depletion compared to the other MAGs, with comparatively less domains, a pattern consistent with their smaller size (S4C Fig). These MAGs were particularly depleted in chaperone associated domains (PF00004 and PF00226) and in an IQ calmodulin-binding motif (PF00612) involved in protein binding. ARC_232 showed a dramatic depletion of a pentatricopeptide repeats domain potentially involved in RNA metabolism (PF13812). A group gathered PSE_171, ARC_217, ARC_116, SOC_37 and ARC_189, most of them showing the same gene and genome characteristics. Compared to all the other MAGs, ARC_189 was dramatically enriched in domains associated with regulators of chromatin structure, domains containing repeat motifs and chromosome condensation repeats (PF00415, PF13540, PF08238, PF12796, PF13517 and PF00651). ARC_217 and PSE_171 were both enriched in the ATP-binding domain of ABC transporters and cyclins (PF00005 and PF00134) while the latter showed a strong depletion in PT/TIG domains (PF01833), a putative family of transcription factors. On the other hand, ARC_116 displayed a strong enrichment in heat shock transcription factor and chlorophyll a-b binding protein domains (PF00447 and PF00504) while SOC_37 displayed a significant enrichment in RNA polymerase Rpb1 C-terminal domain (PF05001). Another group consisted of the genomes PSE_253, ARC_267, MED_399 and PSW_256, all exhibiting medium BUSCO completion. Both PSE_253 and ARC_267 showed an enrichment in zinc-finger domain PF00098 while MED_399 was enriched in domains involved in ubiquitination processes (PF00066 and PF13475). Finally, of all the MAGs, PSW_256 was found strongly enriched in reverse transcriptase (PF00078) and transposase IS4 (PF13843, PiggyBac transposon) domains, a pattern that appeared consistent since this genome was the largest.

# References

1. Nelson DR, Hazzouri KM, Lauersen KJ, Jaiswal A, Chaiboonchoe A, Mystikou A, et al. Large-scale genome sequencing reveals the driving forces of viruses in microalgal evolution. Cell Host Microbe. 2021;29: 250-266.e8. doi:10.1016/j.chom.2020.12.005
