# Supplementary material for: Whole-genome scanning reveals environmental selection mechanisms that shape diversity in populations of the epipelagic diatom Chaetoceros
Source: PLoS Biol. 2022 Nov 28;20(11):e3001893. doi: 10.1371/journal.pbio.3001893 (PMC9731442; doi:10.1371/journal.pbio.3001893)
Supplement: Attachment — Submitted filename: Nef-et-al_Response_to_reviewers.pdf [file pbio.3001893.s050.pdf]

Reviewer #1: Marine microbes interactions.

Reviewer #2: Evolution of microplankton.

Reviewer #1: In the article, "Whole-genome scanning reveals selection mechanisms in epipelagic Chaetogeros diatom populations", Nef and coauthors use 11 Chaetoceros MAGs from the Tara oceans dataset and consider distribution and diversity, with a focus on how microdiversity relates to abiotic factors in the environment. This is an interesting study that aims to address quintessential questions in marine microbial ecology: how is diversity created and maintained in a fluid ecosystem with unrestricted connectivity between populations. As is always the case, these authors, like others, are limited by data availability. However, the manuscript is comprehensive and polished and marks a significant contribution to the field. I have a few general questions that I think, if clarified, would improve the manuscript, as well as some minor comments:

Given that Chaetoceros is described in the introduction as an abundant and globally distributed phytoplankton group with more than 239 species, is it surprising that only 11 Chaetoceros MAGs were recovered from the Tara dataset? I understand that the MAGs were not assembled for this particular publication, but it might be worthwhile to comment on this. Why is the number so low? How does it compare to other phytoplankton groups?

Reply: We thank the reviewer for this comment and acknowledge the concern about the number of *Chaetoceros* MAGs when considering the global abundance of the genus. Indeed, MAGs are recovered from assembling billions of metagenomic reads in a non-redundant manner (all MAGs have an average nucleotide identity < 98%), and represent consensus genomic sequences of closely related cells, as described in the original paper (Delmont et al. 2022). Specifically, the metagenome-assembled genomes were built from metagenomic co-assemblies based on geographically separated samples, and not from single samples. The use of co-assemblies is better for improving the global coverage of large genomes, such as eukaryotes, but as a result tends to fail to cover (locally) low abundant taxa or those displaying very high SNV levels, which might be the case for *Chaetoceros*. We invite the reviewer to consult the Delmont et al. paper (https://doi.org/10.1016/j.xgen.2022.100123) in which the authors explain the advantages and limits of their approach in more details:

“We used the 280 billion reads as inputs for 11 metagenomic co-assemblies (6–38 billion reads per co-assembly) using geographically bounded samples (Figure 1; Table S2), as previously done for the Tara Oceans 0.2–3 mm size fraction enriched in bacterial cells. We favored co-assemblies to gain in coverage and optimize the recovery of large marine eukaryotic genomes. However, it is likely that other assembly strategies (e.g., from single samples) will provide access to genomic data our complex metagenomic co-assemblies failed to resolve” (Results and Discussion section ‘A new resource of environmental genomes for eukaryotic plankton from the sunlit ocean’)

“Nevertheless, the approach failed to cover lineages (1) containing very large genomes (e.g., the Dinoflagelates), (2) only found in low abundance, (3) or found to be abundant but with unusually high levels of microdiversity, challenging metagenomic assemblies […] Deeper sequencing efforts coupled with long read sequencing technologies will likely overcome many of these limitations in years to come.” (Results and Discussion section ‘Limitations of the study’)

We expanded the discussion by explaining the advantages and drawbacks of the reconstruction method in the section ‘*Chaetoceros* metagenome-assembled genomes from *Tara* Oceans’:

“The *Chaetoceros* MAGs studied here were generated from *Tara* Oceans metagenomic co-assemblies [46] based on geographically separated samples, which proved better at increasing the global coverage of large genomes but is limited when covering taxa with low local abundance and/or high microdiversity levels. The *Chaetoceros* genus was notably the best represented of all diatom genera (11 out of 52 diatom MAGs) [46], in agreement with its broad distribution and abundance patterns. Future studies involving increased sequencing effort coupled with innovative assembly strategies, such as automated MAG recovery workflows [80], will likely improve the access to new resources for this genus and other diatoms in the near future.”

I suggest including the recently published Chaetoceros genome mentioned in the discussion in Figure 1, so readers can quickly grasp how these MAGs compare to a genome from the same genus. It would also be helpful to provide more context regarding how closely related the model diatoms and Chaetoceros are: is there reason to suspect that genomes from these groups should have similar size, GC content, etc.?

Reply: We added the *Chaetoceros tenuissimus* NIES-3715 genome in the information displayed in Fig. 2 (former Fig. 1) regarding genome size and BUSCO completion, and in the G+C content shown in S1A Fig. As the gene sequences of this genome are not available, we unfortunately could not add this information in Fig. 2C-D nor for the phylogeny. We nonetheless added a few words about comparing the MAGs with the *C. tenuissimus* genome (Results section 'Description and comparative analysis of *Chaetoceros* MAGs'):

"We first performed a comparative analysis of 11 *Chaetoceros* MAGs (see S1 Table for details on their names) previously assembled from *Tara* Oceans metagenomic co-assemblies [46]. The MAGs displayed genome sizes ranging from 10.6 (ARC_232) to 44.4 (PSW_256) Mbp that are the same order of magnitude as the genomes of the model diatoms *Chaetoceros tenuissimus*, *T. pseudonana* and *P. tricornutum* (Fig 2A)."

"The average percentage of G+C ranged from 39% (ARC_267) to 44% (SOC_37), which is lower than those of *T. pseudonana* and *P. tricornutum*, but in line with that of *C. tenuissimus*, with a global decreasing percentage of G+C from first to third position in the codons (S1A-B Figs). Overall, *T. pseudonana* exhibited genomic characteristics closer to the MAGs and *C. tenuissimus* genome compared with *P. tricornutum*, which is consistent with *Chaetoceros* and *Thalassiosira* genera being classified as diatoms with centric symmetry."

For the BUSCO results, please indicate how many BUSCOs were in the set (n=303?, the number included changes with version). How do BUSCO results turnout when you use the stramenopiles lineage instead of eukaryota? I would also recommend providing the more complete BUSCO results (Complete Single-copy, complete duplicated, fragmented, and missing) as these metrics may be especially important for evaluating MAGs.

Reply: We thank the reviewer for these suggestions. We have now added more details about the number of BUSCOs in the set (n = 255) in Fig 2 (former Fig 1) legend and in the Materials and Methods section 'Genomic resources', as well as a detailed summary as S2 Table. At first, we thought of using the 'alveolata_stramenopiles' reference dataset, but these contained 234 BUSCOs, which we think might be a consequence of including oomycetes and labyrinthulids that are quite divergent from diatoms. We therefore decided to consider the 'eukaryote' database as a more comprehensive and relevant database for the phylogenetic reconstruction, including a broad spectrum of eukaryotes.

Regarding the phylogenetic reconstruction - I have trouble seeing that the MAGs ARC_189 and PSE_253 resolved at the level of C. dichaeta. Based on the tree in the figure, you could just as well say that they resolved at the level of Thalassiosira. Is it possible to construct another tree with more diatom references and a more closely related outgroup to better resolve which taxa these two MAGs are more closely related to? Rather than buscos, you could use single copy orthologs from an orthofinder (or similar) run and then build a tree with more concatenated genes. Alternatively, other methods may be used to better place these two MAGs, but as is, it is difficult for a reader to be confident that they are in the genus Chaetoceros.

Reply: We appreciate the reviewer's concern, and addressed it by conducting an additional analysis using Orthofinder on the 34 species that were initially used to build the BUSCO phylogeny. For this, a total of 846 orthogroups with at least 50% of species having single-copy genes in any orthogroup were considered. The way the MAGs are grouped with the *Chaetoceros* references is globally similar to the BUSCO phylogeny, but with the *Chaetoceros* genus being rather well-defined in one clade, except again for *C. dichaeta*. We added this figure as Supplementary S3 Fig and believe that it will make the MAG positions clearer to the readers. A brief analysis was added to the Results section 'Description and comparative analysis of *Chaetoceros* MAGs':

"We then evaluated the relatedness of the *Chaetoceros* MAGs between one another and with respect to other taxa based on a concatenated tree of 34 taxa for 83 single-copy nuclear genes (total 42,525 amino acids) across the eukaryotic tree of life (Fig 3B). An additional tree was built with the 34 taxa as input to identify their orthogroups based on protein sequences (n = 846 orthogroups) (S3 Fig). For both analyses, we obtained a relatively good phylogeny of the taxa, with a monophyly of the diatoms. As observed previously, MAGs from a close geography did not appear to resolve together. In accordance with the ANI/AAI patterns, three MAG pairs resolved together with high support values, namely ARC_189 and PSE 253, ARC_217 and PSE_171, and ARC_267 and PSW_256 (Figs 3B and S3). Both ARC_189 and PSE_253 MAGs resolved somewhat differently in the clade, which may suggest that they belonged to the *Phaeoceros* subgenera. Conversely, the 9 other MAGs resolved in the same clade as *C. affinis*, *C. curvisetus* and *C. debilis*, indicating closeness to the *Hyalochaete* subgenera."

We therefore modified the Materials and Methods section 'Phylogeny and identification of orthogroups':

"Two analyses with the OrthoFinder [130] software were conducted to identify the orthogroups: a first one using the protein sequences from the MAGs (available at https://www.genoscope.cns.fr/tara/#SMAGs) and the 23 other taxa, in which 846 orthogroups including at least 50% of species having single-copy genes in any orthogroup were used as input to build a species tree in iTOL; and a second one considering only the *Chaetoceros* MAGs to identify their orthologous genes."

Figure 3, B - it is not very clear what is being correlated here - relative abundance at each station? The caption could use a little more detail. Also, it appears that most of the pairs are negatively correlated, maybe -0.25? It would be helpful if the far end of the scale bar was labeled. Finally, Pearson's rho can only asses linearly related data, I would recommend using Spearman's correlation coefficient in this case.

Reply: We agree with the reviewer that Spearman's correlation was best suited for this analysis, and modified Fig 4B (former 3B) accordingly. The results displayed patterns similar to the former ones using the Pearson correlation metric.

I'm generally wondering about time frames for diatom evolution - is stratification a prolonged enough phenomenon to cause genetic differentiation, as is discussed regrading the station TARA_194? Is it also possible that genetic differentiation occurred in two different water masses that are temporarily interleaved? With only two depths and one time-point, it is difficult to make conclusions here?

Reply: We acknowledge the reviewer's concern. It is indeed difficult to make precise conclusions on the timescale of genetic differentiation between populations without any temporal reference. It seems indeed plausible that Pacific populations that were significantly distinct genetically from the others may have been carried into the Arctic, hence the elevated F_{ST} values observed when compared with other stations.

Possible explanations for this particular genetic differentiation pattern have been added to this part of the manuscript, which now reads:

Results section 'Analysis of *Chaetoceros* population structure': "As evidenced by a pairwise F_{ST} of 0.14, both TARA_194 depths appeared to have moderate genetic differentiation among one another. This result suggested that this *Tara* station potentially harboured two sub-populations. Examining the metadata associated with this station revealed that the DCM was sampled 30 m deeper (35 m) than the surface (5 m) (S6 Table), with distinct patterns of oxygen concentration and salinity between the depths as well as a phosphate enrichment at the DCM. One explanation could be that the population of TARA_194 is genetically distinct from the others due to the fact that the Pacific water mass is temporarily interleaved with those more specific to the Arctic. However, in the absence of a temporal reference it is difficult to conclude."

Discussion section 'Genetic differentiation among closely related *Chaetoceros* populations is correlated with different environmental variables': "In particular, ongoing speciation of the ARC_116 population located at station TARA_194, particularly at the DCM, might be a reason explaining why we observed a dramatic number of SNVs, leading us to exclude this station in order to perform a more conservative study when identifying genes under selection. Indeed, this indicates unequal gene flow among the populations and suggests a metapopulation structure consisting of populations of populations,

as has been described for the diatom *D. brightwellii* [104]. This observation might also stem from a mix of Pacific and Arctic populations coming from intermixed water masses. However, this difference in genetic structure of the ARC_116 population at station TARA_194 was not observed in other populations present at this particular station, such as for SOC_37."

Figure 4B - the values should be moved above the bars as they are difficult to read as is
Figure 4E - a map of the regions of the Arctic ocean included in the analysis would be helpful here

Reply: We thank the reviewer for these suggestions. We modified Figures 5B (former 4B) and S9 (former S8) for readability and added a map with the Arctic Ocean regions highlighted as Fig 5F.

Examining the correlation between abiotic parameters and population structure -
How were environmental factors normalized? Log scaled or z-scored?

Reply: The environmental factors were z-score normalized. This has been added in the Materials and Methods section 'Estimation of variance partitioning'. The sentence now reads:

"As an input dataset for abiotic parameters, median values of different environmental parameters were extracted from the PANGAEA database [58–60] and for each sampling site, namely oxygen, salinity, and temperature, as well as concentrations of ammonium, iron, nitrate, nitrite, phosphate and silicate. Environmental variables were z-score normalized."

Were environmental parameters correlated? How did you test for multicolinearity? What was the the variance inflation factor of the model?

Reply: In accordance with the reviewer's questions, we performed different analyses:

1- A correlation analysis on the environmental variables to test for autocorrelation. This highlighted indeed that some variables were correlated (see Fig S27A). After having removed ammonium and adding together nitrate and nitrite to reduce redundancy, we still observed some level of correlation among the environmental parameters (see Fig S27B).
2- We therefore performed a multicolinearity analysis using the matrices involving F_{ST}, Euclidean distances and environmental variables. This showed rather elevated variance inflation factors between the F_{ST}, nitrate & nitrite, and iron matrices in our dataset, despite our efforts to normalize the data and reduce their interdependency patterns.

As all these nutrients are deemed essential for shaping diatom population growth, we chose to keep them for the variance partitioning analyses and added one figure in Supplementary Information. We therefore toned down some of our conclusions and discussed them more cautiously. The manuscript now reads:

Materials and Methods section 'Estimation of variance partitioning': "Correlation between variables was inspected with the R package 'corrplot' which highlighted several correlated parameters (S27A Fig). We therefore removed ammonium and added together nitrate and nitrite into one variable called "Nitrate_Nitrite" to avoid redundancy. The resulting correlation plot still showed significant correlation patterns between phosphate, silicate and Nitrate_Nitrite, and between the latter and iron (S27B Fig). We further analysed the multicolinearity among variables using the R package 'performance' [143], which confirmed elevated (≥ 10) variance inflation factors between the F_{ST}, Nitrate_Nitrite and iron matrices in our dataset. We however chose to keep these variables as all of them were shown to significantly influence diatom growth in the environment."

Results section 'Examining the correlation between abiotic parameters and population structure': "We must however point out that the model error reached 36%, indicating that the model does not apply well to the populations of this particular MAG and that the predictions are to be taken with caution. […] Indeed, a closer investigation of the environmental variable colinearity degree showed that despite our normalization efforts some of them were correlated (see Materials and Methods)."

Discussion section 'Genetic differentiation among closely related *Chaetoceros* populations is correlated with different environmental variables': "Here, although the linear mixed model we applied allowed good predictions for two out of the three MAGs investigated, preventing us from supporting all the variance partitioning results, we observed a correlation of genetic differentiation with phosphate, silicate and iron concentrations in *Chaetoceros* species, in addition to a correlation between temperature and genetic differentiation in SOC_37 populations. As mentioned previously, increasing the number of samples investigated by improving MAG assembly and recovery from the environment would likely improve the statistical robustness of the model."

The lack of statistical significance as determined by Mantel tests may reflect the number of parameters being tested. If only one variable from each group of covarying parameters is included, the test could have more power.

Reply: Here, the Mantel test analyses were conducted independently on each environmental parameter, for each MAG, as shown in the Supplementary Figures S17-S19. We added more details in the Materials and Methods section 'Estimation of variance partitioning' for clarity:

"Mantel tests from the 'vegan' R package [144] were applied to verify the results independently on each environmental variable."

How was geographic distance computed? As the crow flies or based on oceanographic connectedness? Were currents taken into account? I suspect that it was as the crow flies, but I wonder if a more oceanographic distance would be more significant, particularly because it was mentioned earlier in the manuscript that longitude was more correlated with SNVs than latitude and this was attributed to strong oceanic currents in the arctic.

Reply: We thank the reviewer for pointing out ways to improve the analysis by taking oceanic currents into account. In fact, we aimed initially to use Lagrangian distances modeled from the global drifter program as a proxy of travel time. However, the data were very sparse due to poor coverage and few observations in the Arctic Ocean. Using travel times instead of Euclidean distances to compute geographic distances is definitely something that we will target in the future, when datasets with sufficient reliability for the Arctic will be available.

Minor comment: In the introduction, I would support changing "superior trophic levels" to "higher trophic levels".

Reply: This has been modified in the introduction. The sentence is now as follows:

"About half of primary productivity on Earth is supported by aquatic phytoplankton, a phylogenetically diverse group of photosynthetic organisms composed of eukaryotic algae and cyanobacteria that provide essential ecosystem services, from nutrient cycling and CO_2 regulation to sustaining higher trophic levels as the base of marine food webs [1–3]."

Reviewer #2: The article from Nef et al. "Whole-genome scanning reveals selection mechanisms in epipelagic Chaetoceros diatom populations" investigates 11 MAGs attributed to the diatom genus Chaetoceros recently published by Delmont et al. (2022). The article presents a glimpse of the probable future of environmental genomics: Using reference genome to extract MAGs from environmental datasets and investigate the ecology of targeted groups in the light of their molecular properties.
I must say that although I have experience in metabarcoding, I have none in metagenomics and I am not well placed to criticize the technical part of the work because I never did it myself. I represent more the target audience from this type of study and I will comment it from this point of view.
The article reads well and I cannot do any critics on the content. I had some reservation when reading some results (e. g., Weak correlation between geography and population structure, non-significant mantel tests), but these points are addressed in the discussion. The text is generally dense, which makes sometimes difficult to absorb all the information, but I appreciate this type of reading and the sections are articulated logically. There are a few things that I would like to ask the author to add or clarify but this is only minor.

I did not understand why the authors worked with 11 MAGs "only". I know that it is a nice feat but I would like to know why there is not more (or less) genomes. Is it because of the coverage of the data? Or the authors limited themselves to the more dominant/complete MAGs? Maybe I missed the explanation but I feel the information is currently missing. If the information is provided in Delmont et al. (2022), I feel it should be provided in the present paper nonetheless.

Reply: We thank the reviewer for this comment and for seeing value in our methodology. We acknowledge the concern about the number of *Chaetoceros* MAGs when considering the global abundance of the genus. As stated in the response to Reviewer 1, MAGs were recovered from assembling billions of metagenomic reads in a non-redundant manner (all MAGs have an average nucleotide identity < 98%), and represent consensus genomic sequences of closely related cells. Here, the metagenome-assembled genomes were built from metagenomic co-assemblies based on geographically separated samples, and not from single samples. The use of co-assemblies is better for improving the global coverage of large genomes, such as eukaryotes, but as a result tends to fail to cover (locally) low abundant taxa or those displaying very high SNV levels, which might be the case for *Chaetoceros*.

We invite the reviewer to consult the Delmont et al. paper (https://doi.org/10.1016/j.xgen.2022.100123) in which the authors explain the advantages and limits of their approach in more detail:

“We used the 280 billion reads as inputs for 11 metagenomic co-assemblies (6–38 billion reads per co-assembly) using geographically bounded samples (Figure 1; Table S2), as previously done for the Tara Oceans 0.2–3 mm size fraction enriched in bacterial cells. We favored co-assemblies to gain in coverage and optimize the recovery of large marine eukaryotic genomes. However, it is likely that other assembly strategies (e.g., from single samples) will provide access to genomic data our complex metagenomic co-assemblies failed to resolve” (Results and Discussion section ‘A new resource of environmental genomes for eukaryotic plankton from the sunlit ocean’)

“Nevertheless, the approach failed to cover lineages (1) containing very large genomes (e.g., the Dinoflagelates), (2) only found in low abundance, (3) or found to be abundant but with unusually high levels of microdiversity, challenging metagenomic assemblies […] Deeper sequencing efforts coupled with long read sequencing technologies will likely overcome many of these limitations in years to come.” (Results and Discussion section ‘Limitations of the study’)

We expanded the discussion by explaining the advantages and drawbacks of the reconstruction method in the section ‘*Chaetoceros* metagenome-assembled genomes from *Tara* Oceans’:

“The *Chaetoceros* MAGs studied here have been generated from *Tara* Oceans metagenomic co-assemblies [46] based on geographically separated samples, which proved better at increasing the global coverage of large genomes but is limited when covering taxa with low local abundance and/or high microdiversity levels. The *Chaetoceros* genus was notably the best represented of all diatom genera (11 out of 52 diatom MAGs) [46], in agreement with its broad distribution and abundance patterns. Future studies involving increased sequencing effort coupled with innovative assembly strategies, such as automated MAG recovery workflows [80], will likely improve the access to new resources for this genus and other diatoms in the near future.”

I feel the authors should supply a graphic support to introduce the Chaetoceros genus (Ideally Figure 1) to the audience. Specifically, I feel it would be useful to provide the occurrence of OTUs from the metabarcoding dataset from the TARA Oceans to show the biogeographic occurrence of Chaetoceros, together with the number of OTUs ascribed to this genus (Compared to the number of taxa in Algaebase). This will provide an appreciation of the difference of representation of the same genus between morphological, metabarcoding and metagenomics datasets. Also, I think that providing light microscopy images and eventually an SEM from Chaetoceros would be useful for the non-diatom experts, as well as a map with the a polar projection (like in the figure S14) next to classical projection to appreciate better the geographical organisation in the arctic.

Reply We acknowledge that the manuscript could benefit from more details about the *Chaetoceros* genus, as it is not yet a model organism. As such, a map illustrating *Chaetoceros* global distribution using metabarcoding data (V9 18S rDNA) from the *Tara* Oceans expeditions has been added as Fig. 1. A map with polar projection of the Arctic Ocean region has also been added to Fig. 5 (former Fig. 4).

I would remove the mention of the FST from the abstract, as it is not yet defined and not every reader might know what it means.

Reply: We agree and have removed the details of F_{ST} values from the abstract.

I support the publication of this manuscript but as stated above, I cannot criticize to methodological part of the work and I hope this will be covered by other reviewers.
